# TET proteins regulate T cell and iNKT cell lineage specification in a TET2 catalytic dependent manner

**DOI:** 10.3389/fimmu.2022.940995

**Published:** 2022-08-05

**Authors:** Tarmo Äijö, Dimitris Theofilatos, Meng Cheng, Matthew D. Smith, Yue Xiong, Albert S. Baldwin, Ageliki Tsagaratou

**Affiliations:** ^1^ Lineberger Comprehensive Cancer Center, University of North Carolina at Chapel Hill, Chapel Hill, NC, United States; ^2^ Curriculum in Genetics and Molecular Biology, University of North Carolina at Chapel Hill, Chapel Hill, NC, United States; ^3^ Department of Genetics, University of North Carolina at Chapel Hill, Chapel Hill, NC, United States; ^4^ Department of Microbiology and Immunology, University of North Carolina at Chapel Hill, Chapel Hill, NC, United States

**Keywords:** TET proteins, Th-POK, DNA Methylation, 5hmC, lineage specification

## Abstract

TET proteins mediate DNA demethylation by oxidizing 5-methylcytosine to 5-hydroxymethylcytosine (5hmC) and other oxidative derivatives. We have previously demonstrated a dynamic enrichment of 5hmC during T and invariant natural killer T cell lineage specification. Here, we investigate shared signatures in gene expression of *Tet2/3* DKO CD4 single positive (SP) and iNKT cells in the thymus. We discover that TET proteins exert a fundamental role in regulating the expression of the lineage specifying factor Th-POK, which is encoded by *Zbtb7b*. We demonstrate that TET proteins mediate DNA demethylation - surrounding a proximal enhancer, critical for the intensity of Th-POK expression. In addition, TET proteins drive the DNA demethylation of site A at the *Zbtb7b* locus to facilitate GATA3 binding. GATA3 induces Th-POK expression in CD4 SP cells. Finally, by introducing a novel mouse model that lacks TET3 and expresses full length, catalytically inactive TET2, we establish a causal link between TET2 catalytic activity and lineage specification of both conventional and unconventional T cells.

## Introduction

The TET family of proteins consists of three members: TET1, TET2 and TET3 that are Fe2^+^ and O_2_ dependent deoxygenases, with the enzymatic capacity to oxidize 5-methylcytosine (5mC) to 5-hydroxymethylcytosine (5hmC) (1) and downstream additional oxidized modified cytosines (oxi-mCs), 5-formylcytosine (5fC) and 5-carboxylcytosine (5caC) (2). Notably, TET proteins mediate active DNA demethylation and promote gene expression (3). In addition, all the above oxi-mCs can also exist as stable epigenetic marks, that can affect the binding and the activity of transcription factors that preferentially recognize specific modified or unmodified states of cytosine (4–7). TET1 and TET3 share a CXXC DNA binding domain, whereas TET2 lacks a DNA binding domain, and thus cannot directly bind to the DNA (3). Interestingly, loss of TET proteins in various cell types did not reveal a global gain of DNA methylation (8–10). Instead, TET proteins regulate DNA demethylation in a focal manner, mainly at genomic loci that control cell specific genes (8, 11). The precise recruitment of TET proteins to these loci is mediated *via* interaction with transcription factors (11–15). We have previously demonstrated that 5hmC exhibits a dynamic intragenic distribution during T cell lineage specification (16) in the thymus as well as during T helper cell lineage choice (16, 17). Moreover, 5hmC enrichment correlates with high gene expression of T cell specific genes and is frequently detected in active enhancers (16). Paradoxically, concomitant deletion of both TET2 and TET3, which are most highly expressed in T cells, in the double positive (DP) cell stage demonstrated that TET proteins are dispensable for CD4 cell lineage specification (8). Similar findings were reported for simultaneous deletion of TET1 and TET3 (18).

T cell development in the thymus is a tightly regulated, highly complex process (19). DP cells that react strongly against antigens are eliminated by negative selection, whereas DP cells which react below an optimal threshold to antigens are eliminated by a process known as death by neglect (19, 20). Thus, 90% of DP cells are eliminated and only those that demonstrate optimal reaction against antigens are positively selected, signaled to survive and further differentiate to give rise to single positive (SP) CD4 and CD8 cells (21). Specifically, DP thymocytes that are positively selected through major histocompatibility complex (MHC) class II antigen presentation give rise to CD4 cells, whereas the DP cells that recognize peptides through MHC class I molecules give rise to CD8 SP cells (20). GATA3 is required for the CD4 lineage specification (22, 23), and induces Th-POK expression, which is indispensable for CD4 differentiation and maturation (24). Th-POK is the specifying factor of the CD4 lineage (25, 26), consists of four zinc fingers and a BTB domain (bric-a-brac, tram-track, broad complex) and belongs to the large Zinc finger and BTB domain containing (ZBTB) family of proteins (26). Deletion of Th-POK results in redirection of MHC class II restricted CD4 cells to CD8 cells, whereas overexpression of Th-POK impair CD8 differentiation and redirects MHC class I restricted thymocytes to differentiate towards the CD4 lineage (25, 26). Recently, the precise binding sites of Th-POK have been revealed (27) which indicate that Th-POK can bind to the DNA of CD4 as well as CD8 specific genes, such as *Cd4*, *Runx3*, *Cd40lg* and *Zbtb7b*, which encodes Th-POK (27). Mature CD4 cells will migrate from the thymus to peripheral organs. Based on the cytokine milieu and environmental cues naïve CD4 cells will give rise to helper lineages that express distinct lineage specifying factors and secrete specific cytokines (28, 29).

DP thymocytes that are selected through the CD1d MHC class I like molecule give rise to invariant natural killer T (iNKT) cells, a unique subset of innate-like T cells (30). iNKT precursor cells rapidly upregulate the ZBTB transcription factor promyelocytic lymphocyte zinc finger (PLZF), which endows them with effector properties (31, 32). They express an invariant Va14 chain and a limited number of Vβ chains and they recognize lipids (30). Thymic iNKTs exhibit an antigen experienced phenotype that endows them with the capacity to potently secrete large quantities of cytokines, including those that are typically secreted by helper T cells (33, 34). Specifically, iNKT cells that upregulate GATA3 acquire phenotypical characteristics reminiscent of the T helper 2 (Th2) cells, secrete interleukin 4 (IL-4) and are characterized as NKT2 cells (35). Thymic iNKT cells that express RORγt and secrete IL-17 are known as NKT17 cells (35). Finally, an additional iNKT subset in the thymus has been described that upregulates T-bet, potently secretes IFNγ and is known as NKT1 subset (35). Notably, NKT1 cells maintain the capacity to also secrete IL-4 (36).

The transcriptional circuitries that shape conventional and unconventional T cell identity and lineage specification as well as lineage integrity (37–42) have been extensively studied. The individual contribution of the transcription factors that regulate lineage commitment and maintenance has been dissected in detail, by generating mouse models that specifically lack the expression of the factor of interest at distinct stages of T cell development and differentiation. Recent studies have indicated a critical role for epigenetic regulators in the process of T cell and iNKT cell development (42–44). However, the precise epigenetic mechanisms that regulate the optimal expression and function of transcription factors to orchestrate the process of thymic lineage specification have not been elucidated.

Deciphering the roles of TET proteins and 5hmC in shaping T and iNKT cell lineage fate and maturation is of paramount importance. Proper maturation and lineage specification ensure optimal immune response against pathogens as well as tumor cells (36, 45). In this study, we seek to understand how TET proteins, and in particular TET2 and TET3, which are most highly expressed in T cells (46, 47), impact the gene expression program of CD4 SP cells. Then, we identify shared signatures among *Tet2/3* DKO iNKT cells and CD4 SP cells in the thymus. We reveal that concomitant loss of TET2 and TET3 reduces significantly Th-POK expression due to increased DNA methylation in the *Zbtb7b* locus in both iNKT cells and CD4 SP cells, revealing a conserved role of TET2 and TET3. Specifically, we identify gain of methylation at the proximal enhancer and at site A, where GATA3 binds to regulate Th-POK expression. Finally, we ask if the observed defects in iNKT and T cell lineage specification are exclusively regulated by TET2 catalytic dependent activity or if TET2 exerts additional, catalytic independent roles. We were inspired to pursue this question by our previous findings that TET3 deficiency is sufficient to increase NKT17 frequency, but did not result in the striking expansion that we observed for the *Tet2/3* DKO iNKT cells (8). Recent studies suggested that TET2 can impact lymphoid cells in a catalytic independent manner (48). Moreover, TET2 has been reported to regulate inflammatory phenotypes in macrophages by mechanisms that are not related to its enzymatic activity (49). Here, we introduce a novel mouse model that lacks TET3 and expresses full length TET2, which is catalytically inactive due to a point mutation that compromises iron binding. Our data establish a fundamental role of the catalytic activity of TET2 that in cooperation with TET3 shapes conventional and unconventional lineage specification in the thymus.

## Methods

### Mice

All mice were bred and maintained under specific pathogen-free conditions at University of North Carolina (UNC) Genetic Medicine building, in a facility managed by the Division of Comparative Medicine at UNC Chapel Hill. The experimental procedures were approved by the UNC Institutional Animal Care and Use Committee. C57BL/6 (B6) (stock number: 000664) and B6.SJL-CD45.1 (stock number: 002014) were initially purchased from Jackson Laboratories and were bred and maintained in our facility. *Tet2* ^-/-^ mice (mentioned *Tet2* KO hereafter) (46) were initially generated by the Rao lab and were purchased from Jackson laboratory (stock number: 023359). *Tet3* ^flx/flx^ (50) mice were generated in the Rao lab (and are currently available from Jackson laboratory, stock number: 031015). *Tet3* ^flx/flx^ CD4Cre (mentioned *Tet3* KO hereafter) (8) mice and *Tet2* ^-/-^ *Tet3* ^flx/flx^ CD4Cre (mentioned *Tet2/3* DKO hereafter) (8) mice have been previously described. *Tet2* ^H1795R^ mice (referred to as *Tet2* ^CD^ hereafter) were designed and generated by MC, MS, YX and ASB at the UNC Animal Models Core (51). For the immune-phenotyping experiments both male and female mice were used. For genome-wide sequencing experiments, we used sex and age-matched mice. When feasible, both male and female mice were used as indicated in the specific methods sections. The control and the TET mutant mice were analyzed between 21-30 days old, during this time the mice were healthy (8). The genotypes of the mutant mice were validated by PCR genotyping. Genomic DNA was isolated using Phire Animal Tissue Direct PCR kit (Thermo scientific, cat no F-140WH), based on the manufacturer’s guidelines. PCR amplification of DNA fragments was performed using the Phire DNA polymerase (Thermo scientific) and specific primers using Biorad T100 or Biorad C1000 Touch thermocyclers. PCR products were run in a 3% agarose gel and were visualized using SYBR safe (Invitrogen, Thermo Scientific) staining using an Axygen Gel documentation system. For the *Tet2^CD^* mice, DNA was isolated as described above and amplified using Phusion High Fidelity DNA polymerase (NEB). The sequence of the primers used for the amplification is:

Tet2-H1795R-F: GTGCTTCCAGGGTTTAATCATG

Tet2-H1795R-R: GTCTGATCCATTCTTTCCGCA

PCR products were submitted for purification and sequencing at Eton Bioscience. The sequence of the primer used for sequencing was Tet2-H1795R-Seq: GCAGCAGTCAGGAGAAGCAG. Sequencing results were analyzed to distinguish heterozygotes from homozygotes.

### Flow cytometry

Staining of thymocytes, was performed directly *ex vivo*. Briefly, mice were euthanized and thymi were harvested. Single-cell suspensions were prepared by dissociating the organs using a 70 um cell strainer (Falcon). For surface stainings, cells were stained in FACS buffer (PBS containing 2% FBS). Dead cells were excluded by using fixable viability dye (eBioscience). To perform multiparameter Flow cytometry, antibodies were used conjugated with fluorophores: Anti-mouse CD4^AF488^ (clone: RM4-5), anti-mouse CD8^BV650^ (clone: 53-6.7), anti-mouse TCR-β^PERCP/Cy5.5^ (clone:H57-597), CD44^PERCP/Cy5.5^ (clone: IM7) were all from Biolegend. The antibodies were used in a dilution 1:200. aGalactosyl-ceramide loaded tetramer conjugated with PE or BV421 was obtained from NIH tetramer Core and was used for stainings in a dilution 1:400. For intracellular staining, cells were washed three times with FACS buffer and were fixed and permeabilized using the Foxp3 transcription factor staining kit (eBioscience) according to the manufacturer’s instructions. The fixation step was performed overnight. Antibody for transcription factor Th-POK^Alexa647^ (clone: T43-94) was purchased from BD Pharmingen and was diluted in 1X Permeabilization buffer (diluting the 10X permeabilization buffer available in the Foxp3 transcription factor staining kit) in a dilution 1:20 according to the instructions. PLZF^A647^ (clone: R17-809), RORγt (clone: Q31-378) conjugated either with CFP594 or BV421 and GATA3 (clone: L50-823) conjugated with PE were all from BD Pharmingen. PLZF and RORγt were diluted in 1X Permeabilization buffer in a dilution 1:100. GATA3^PE^ was diluted in 1X Permeabilization buffer in a dilution 1:5 (as recommended by the manufacturer). EOMES^EF660^ (clone: Dan11mag) was purchased from eBioscience and was used in a dilution 1:100.

Samples were analyzed by Flow Cytometry using a BD LSRFortessa analyzer (BD Biosciences) or a Novocyte 3005 (ACEA, Agilent). Data were acquired using BD FACSDiva software or NovoExpress (Agilent) software respectively. The data were analyzed and FACS plots were generated using FlowJo (Treestar).

### Cell enrichment & flow cytometry activated sorting

Total thymocytes were depleted of CD24^hi^ cells by staining with biotinylated mouse anti-CD24 (clone:M1/69) purchased from Biolegend. Unwanted cells were depleted by binding to mouse streptavidin magnetic beads (anti-mouse Rapidspheres, cat no 19860, Stemcell Technologies) according to the manufacturer’s instructions. Enriched cells were stained with viability dye (eBioscience) to distinguish live from dead cells. Cells were stained with aGalactosyl-Ceramide loaded tetramer (conjugated with PE, obtained from NIH tetramer core), TCRβ (clone:H57-597) conjugated with PERCP/Cy5.5 (Biolegend), anti-mouse CD4^AF488^ (clone:RM4-5), CD8 (clone:53-6.7) conjugated with Brilliant Violet 650 or APC. For the thymic differentiation related studies, live CD4^+^, CD8^-^, TCRβ^+^, tetramer^-^ cells were sorted and used in downstream applications. The purity of the samples after sorting was >98%. The cells were sorted using an Aria Sorter (Becton Dickinson).

### RNA sequencing

Live, CD24^low^ enriched cells CD4^+^, TCRβ^+^, aGalCerCd1d tetramer^-^, CD8^-^ cells were sorted in a purity higher than 98%. RNA was isolated using the micro RNeasy plus kit (Qiagen). RNA was quantified using HS RNA kit (Invitrogen) in a Qubit 4 Fluorometer (Invitrogen). RNA integrity was assessed in a Bionalyzer (Agilent) using RNA 6000 pico kit (Agilent, cat no: 5067-1513). The RNA that was used for downstream steps had a RIN value of 9 or higher. Libraries were prepared using the SMARTseq kit v4 Ultra Low Input RNA kit for sequencing (Clontech, cat no: 634888) and Nextera XT (Illumina). Sequencing was performed using Hiseq 4000 (Illumina), at the UNC High Throughput Sequencing Facility. 3 male control (wild type) and 2 male *Tet2/3* DKO mice were used.

### Template capture hybridization sequencing (CATCH-seq)

In order to analyze cytosine methylation status of *Zbtb7b* locus in control (wild type) and *Tet2/3* mutant CD4 SP cells, DNA was isolated from FACS sorted CD4 SP cells using PureLink genomic DNA mini kit (Invitrogen). DNA was isolated following the manufacturer’s instructions and was eluted in 50 ul molecular biology grade water. A minimum of 1.1 ug of DNA was provided to Biodynami (Huntsville, Alabama) for bisulfite conversion, capture, library preparation and next generation sequencing. Biodynami NGS DNA Library Prepkit (cat no 30023) was used and methylated adapters were utilized. Bisulfite conversion was performed with the Epitect Bisulfite kit (Qiagen, catalogue number 59104). For the capture of the mm10 chromosome 3: 89,373,714-89,397,292 a Biodynami Custom Capture kit was developed and used. Sequencing of the generated, barcoded libraries was performed using HiSeq X platform and PE 2x150 reads.

### CUT&RUN

For the CUT&RUN experiments, we used the CUTANA™ ChIC/CUT&RUN Kit (Epicypher, cat no: 14-1048) according to the manufacturer’s instructions with some modifications. Specifically, cells were collected in a V-bottom 96 well plate for handling as previously described (52). We omitted the use of Concanavalin-A (ConA) – coated beads, since ConA is a T cell mitogen. The starting material for each experiment was 500,000 CD4 SP sorted cells. Additionally, the incubation with the antibodies (0.5 ug) was performed at 4°C for 1h. The antibodies used for these experiments are: GATA3 (clone: D13C9) XP from Cell Signaling Technology (CST cat no: 5852) and IgG (Epicypher, cat no: 13-0042).

CUT&RUN libraries were prepared as previously described (53) with modifications (54). Briefly, 3 ng DNA (corresponding to small fractions of DNA) were used for library preparation utilizing NEBNext Ultra II DNA Library Prep Kit (NEB, cat no: E7645). The DNA was end-repaired at 20°C for 30 minutes, followed by an incubation at 50°C for 60 minutes. The adaptor was diluted to 1:12.5 for adaptor ligation and added to the end-repaired products, followed by an incubation at 20°C for 15 minutes. Then, the USER enzyme was added to the ligation mixture and the samples were incubated at 37°C for 15 minutes. To clean up the adaptor-ligated DNA, 1.75 volume of AMPure XP beads (Beckman) was added to the ligation reaction. The adaptor-ligated fragments were amplified using the following PCR conditions: 98°C for 45 sec, 12 cycles of 98°C for 15 seconds, 60°C for 10 seconds and a final extension step: 65°C for 5 minutes. For the PCR amplification, we used the NEBNext Multiplex Oligos for Illumina (Dual Index Primers Set2, NEB, cat no: E7780S). After amplification, libraries were purified with 1.0x volume of AMPure XP beads (Beckman). The DNA concentration was measured using the double stranded (ds) DNA High Sensitivity (HS) assay in a Qubit 4 Fluorometer (Invitrogen). The quality control and the size of the library was determined using High Sensitivity (HS) D1000 Screen Tape assay (Agilent, cat no: 5067-5584), including HS D1000 screen tape and HS D1000 reagents (such as HS D1000 sample buffer and HS D1000 ladder, Agilent, cat no: 5067-5585), in a Tapestation 4150 (Agilent). Libraries were sequenced using Illumina NovaSeq 6000 platform and 50bp PE reads at the Duke Center for Genomic and Computation Biology, of the Duke University Sequencing and Genomic Technologies.

### Purification of proteins

Total thymocytes were isolated from WT, *Tet2/3* DKO, *Tet2* ^CD^ *Tet3* KO, *Tet2* KO, *Tet2* ^CD^ and *Tet3* KO mice. We used both male and female mice. To separate nuclear extracts from cytoplasmic extracts, we used the NE-PER nuclear and cytoplasmic extraction kit (Thermo Scientific, cat no:78833) according to the manufacturer’s instructions. For the purification of protein extracts from sorted CD4 SP cells, isolated from WT and *Tet2* ^CD^ *Tet3* KO mice, we utilized the radioimmunoprecipitation assay (RIPA) buffer. Briefly, FACS sorted CD4 SP cells were washed with ice-cold 1X PBS and then lysed to RIPA buffer (1.0% Igepal CA-630, 0.5% Sodium Deoxycholate, 0.1% SDS, 150mM NaCl, 2mM EDTA, 25mM Tris-HCL pH 7.4) supplemented with protease inhibitors. After a 30 minutes rotation at 4°C, the cell lysates were centrifuged at 10.000g for 10min (at 4°C) and subsequently the supernatant containing the protein extracts was collected. To determine the protein concentration, we used the qubit protein assay kit (Q33211, Invitrogen) and the micro BCA protein assay kit (Pierce, cat no: 23235). Samples were measured in a Qubit 4 fluorometer (Invitrogen) or a Synergy 5 microplate reader (BioTek) respectively. Protein extracts were stored at -80°C.

### Immunoblotting

Prior to immunoblotting, the extracts were diluted in 4xLaemli buffer (Biorad, Cat No: 1610747), containing 10% β-mercaptoethanol, and boiled for 10 minutes. Then, the protein samples were loaded onto 4-20% Mini-PROTEAN TGX Precast Protein Gels (Biorad, cat no: 4568094), subjected to SDS-PAGE electrophoresis, and transferred to PVDF membranes by utilizing the Trans-Blot Turbo Transfer System (Biorad) according to manufacturer’s guidelines. Membranes were blocked with 5% non-fat milk (Biorad) in PBS supplemented with 0.1% Tween (Sigma) and then incubated with primary antibodies against TET2 (dilution 1:1000, CST, cat no: 36449), TET3 (dilution 1:1000, CST, cat no: 99980), Histone H3 (for nuclear extracts, dilution 1:5.000, Sigma, cat no: H0164) and GAPDH (for whole cell extracts, dilution 1:20.000, Sigma, clone: G8795). The HRP-conjugated anti-rabbit (dilution 1:2000, Biorad, cat no: 172-1019) and anti-mouse (dilution 1:3000, Biorad, cat no: 172-1011) secondary antibodies were used. Signals were detected by Pierce ECL Western blotting substrate (Thermo Scientific, cat no: 32209) and proteins were visualized using a ChemiDoc MP imaging system (Biorad). To quantify the Western Blot bands, we used the Image Lab Software (BioRad), while the protein levels of H3 (for nuclear extracts of total thymocytes) or GAPDH (for whole cell extracts of sorted CD4 SP cells) were used for normalization.

### RNA isolation and quantitative PCR

Total RNA was isolated from thymocytes from WT, *Tet2* KO, *Tet2* ^CD^ and *Tet2* ^CD^ *Tet3* KO mice using the RNeasy Plus Mini Kit (Qiagen, cat no:74134) according to the manufacturer’s instructions. Both male and female mice were used. cDNA was synthesized using the iScript Reverse Transcription SuperMix (Biorad, cat no:1708840). qPCR was performed using the iTaq Universal SYBR Green Supermix (Biorad, cat no: 172-5121) on a CFX96 Real-Time System (Biorad). The expression of the target genes was normalized to the expression of *Gapdh*. The relative gene expression levels were determined by the comparative Ct method (ΔΔCt method) according to the CFX Maestro software (Biorad).

### Data statistical analysis

Data were analyzed using Prism software (Graphpad). Unpaired student’s *t* test was applied as indicated. In each figure legend, the indicated p-values are described. Data are mean ± s.e.m. In the graphs, each dot represents a mouse. Unless otherwise indicated the p-value was not statistically significant (p > 0.05). Differences were considered significant when p < 0.05 (^∗^); < 0.01 (^∗∗^); < 0.001 (^∗∗∗^); < 0.0001 (^∗∗∗∗^).

For each experiment, sufficient number of mice was used, to ensure adequate power for our findings. For the phenotyping experiments, mice from different litters and of different sex were evaluated, with reproducible results.

### Graph design

The graph in **Supplementary Figure 1** was designed using Biorender.

### RNA-seq data analysis

Adapter trimming and quality filtering of the sequencing libraries was done using fastp (0.21.0) (55) with the default parameters. The sequencing libraries were mapped against mm10 and the GRCm38.100 transcriptome using STAR (2.7.5a) (56) with the following parameter values: –quantMode GeneCounts. The differential expression analysis was done using DESeq2 (57) based on the read counts per gene produced by STAR. The used threshold for adjusted p-value was 0.01.

### ChIP-seq data analysis

The sequencing libraries were mapped against mm10 using Bowtie 2 (2.4.1) (58) using the default parameters. Reads with identical sequences were filtered and only one was retained for subsequent analysis. The coverage tracks were generated from the samples obtained by pooling the biological replicates using HOMER (4.10) (-norm 1e6) (59).

### CMS-seq data analysis

The CMS-IP and input reads were mapped against mm10 using Bismark (0.22.3) (60). The mapping was done using the Bowtie 2 (2.4.1) (58) backend in the paired-end mode with the following parameter values: -I 0 -X 600 -N 0. The coverage tracks were generated using HOMER (4.10) (makeBigWig.pl -norm 1e6) (59).

### ATAC-seq data analysis

Adapter trimming and quality filtering of the sequencing libraries was done using fastp (0.21.0) (55) with the default parameters. The sequencing libraries were mapped against mm10 using Bowtie 2 (2.4.1) (–very-sensitive -X 2000) (58). Mitochondrial reads were removed after alignment. Additional filtering was done using samtools (1.12) (61) using the following parameter values: -q 30 -h -b -F 1804 -f 2. Reads with identical sequences were filtered and only one was retained for subsequent analysis. The coverage tracks were generated from the samples obtained by pooling the biological replicates using HOMER (4.10) (makeBigWig.pl -norm 1e6) (59).

### CATCH-seq (bisulfite) data analysis

Adapter trimming and quality filtering of the sequencing libraries was done using trim galore (0.6.2) (–paired) [DOI: 10.5281/zenodo.5127899]. The sequencing libraries were mapped against mm10 using Bismark (0.22.3) (60). The mapping was done using the Bowtie 2 (2.4.1) (58) backend in the paired-end mode with the following parameter values: –non_directional. The counts of converted and unconverted cytosines in CpG context were extracted using bismark_methylation_extractor (–bedGraph –counts) (60).

### CUT&RUN data analysis

Adapter trimming and quality filtering of the sequencing libraries was done using fastp (0.21.0) (55) with the default parameters. The sequencing libraries were mapped against mm10 using Bowtie 2 (2.4.1) (–very-sensitive -X 2000) (58). Mitochondrial reads were removed after alignment. Additional filtering was done using samtools (1.12) (61) using the following parameter values: -q 30 -h -b -F 1804 -f 2. Reads with identical sequences were filtered and only one was retained for subsequent analysis. The coverage tracks were generated from the samples obtained by pooling the biological replicates using HOMER (4.10) (makeBigWig.pl -norm 1e6) (59). The peaks were identified from the pooled samples against controls using HOMER (4.10) (findPeaks -style factor). The coverage at the transcription sites was quantified using HOMER (4.10) (annotatePeaks.pl tss mm10 -size 1000 -hist 5 -ghist) (59). The coverage at the peak sites was quantified using HOMER (4.10) (annotatePeaks.pl peaks.txt mm10 -size 1000 -hist 5 -ghist) (59).

### Annotation of regulatory sites across *Zbtb7b* locus

To annotate the distal enhancer and proximal enhancer of *Zbtb7b* locus we utilized sequences of primers described in (62) and we mapped these to the mm10 reference genome (proximal enhancer is chr3:89,385,456-89,385,868 and distal enhancer is chr3: 89,395,980-89,396,394). To annotate site A, where GATA3 has been previously reported to bind, we utilized the sequences described in (24) and mapped them to the mm10 reference genome (chr3:89,382,182-89,382,343).

### Data availability

RNA-seq, CUT&RUN and CATCH-seq datasets have been deposited in the Gene Expression Omnibus (GEO) public repository under the following accession numbers: SuperSeries: GSE206450, RNA-seq data: GSE190230, CUT&RUN: GSE190228, CATCH-seq: GSE190227.

We have not generated unique or novel codes for data analysis for this manuscript. We have provided a detailed data analysis section.

Most of the biological tools (mouse strains) are available at Jackson laboratories and we have provided the relevant stock numbers. The *Tet2* ^CD^ mice are a new strain (51). Requests regarding the *Tet2* ^CD^ mice should be addressed to Dr. Baldwin (albert_baldwin@med.unc.edu). Any other requests should be addressed to Dr. Ageliki Tsagaratou (ageliki_tsagaratou@med.unc.edu).

## Overview of data sets

**Table:** 

Sample	Type	GEO accession	Reference
WT CD4 SP	RNA-seq	GSE190230	Present study
*Tet2/3 DKO CD4 SP*	RNA-seq	GSE190230	Present study
WT CD4 SP	CATCH-seq	GSE190227	Present study
*Tet2/3 DKO CD4 SP*	CATCH-seq	GSE190227	Present study
WT CD4 SP	GATA3 CUT&RUN	GSE190228	Present study
*Tet2/3 DKO CD4 SP*	GATA3 CUT&RUN	GSE190228	Present study
WT iNKT cells	WGBS	GSM1855585 GSM1855586	(8)
*Tet2/3* DKO iNKT cells	WGBS	GSM1855587 GSM1855588	(8)
WT iNKT cells	RNA-seq	GSM2281118 GSM2281119	(8)
*Tet2/3* DKO iNKT cells	RNA-seq	GSM2281122 GSM2281123	(8)
WT immature CD4SP	ATAC-seq	GSM4486874 GSM4486875 GSM4486876	(27)
Th-POK KO immature CD4SP	ATAC-seq	GSM4486877 GSM4486878 GSM4486879	(27)
WT mature CD4 SP	RNA-seq	GSM4486850 GSM4486851 GSM4486852	(27)
WT immature CD4 SP	RNA-seq	GSM4486863 GSM4486865 GSM4486869	(27)
Th-POK Ctrl ChIP	ChIP-seq	GSM4486883	(27)
Th-POK ChIP	ChIP-seq	GSM4486880 GSM4486881 GSM4486882	(27)

## Main

### Altered gene expression program in *Tet2/3* DKO CD4 SP cells

**Figure 1:**
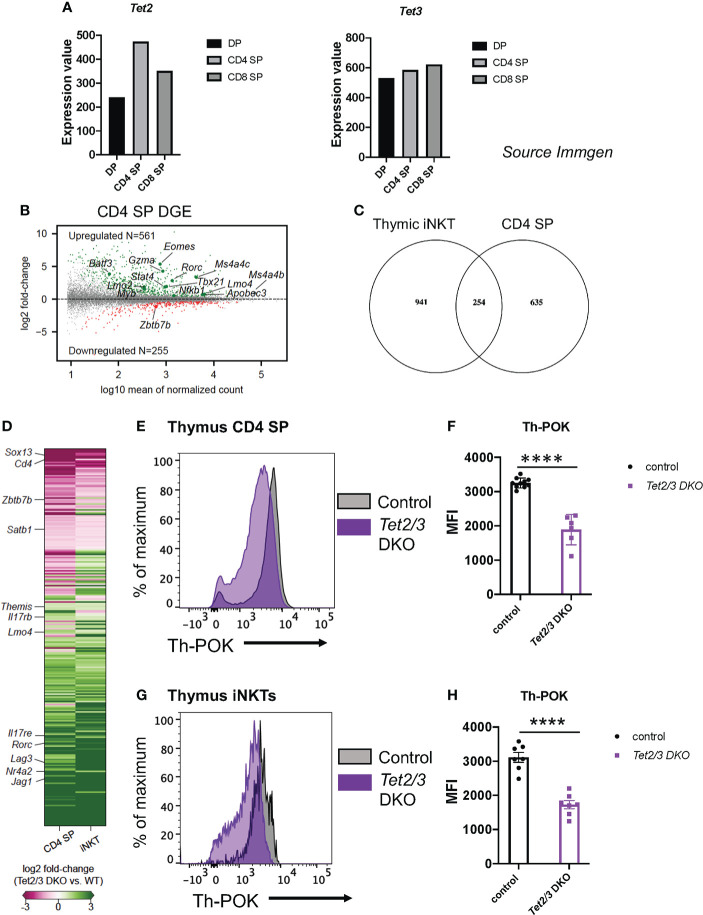
*Tet2* and *Tet3* are upregulated during T cell lineage specification and are required for optimal Th-POK expression. **(A)** Gene expression of *Tet2* (*left*) and *Tet3* (r*ight*) in DP cells, CD4 SP cells and CD8 cells. (Source: Immunological Genome project, Immgen). **(B)** MA plot depicting log_2_ fold-change of gene expression in *Tet2/3* DKO CD4 SP cells compared to control (wild type) CD4 SP cells. 561 were identified to be upregulated (p_adj_ < 0.01, *in green*) in *Tet2/3* DKO SP cells, whereas 255 were downregulated (p_adj_ < 0.01, *in red*). Genes of interest are indicated. n=3 male control (wild type) and 2 male *Tet2/3* DKO mice were used. **(C)** Venn diagram indicating the intersecting genes that are differentially expressed in *Tet2/3* DKO iNKT cells compared to wild type iNKT cells (8) and *Tet*2/3 DKO CD4 SP cells compared to wild type CD4 SP cells. **(D)** Heatmap indicating the log_2_ fold-change of the overlapping genes identified in **(C)**. The scale for evaluating differential gene expression is depicted. **(E)** Representative histogram comparing the protein expression levels of Th-POK in wild type (*gray*) and *Tet*2/3 DKO (*purple*) CD4 SP cells evaluated by flow cytometry. **(F)** Median Fluorescence Intensity (MFI) of Th-POK expression in CD4 SP cells as evaluated in **(E)**. Each dot represents a mouse. WT n=10 (in black), *Tet2/3* DKO n=6 (in purple). For each genotype male and female mice were analyzed with comparable findings. **(G)** Representative histogram comparing the protein expression levels of Th-POK in wild type (*gray*) and *Tet*2/3 DKO (*purple*) iNKT cells evaluated by flow cytometry. **(H)** Median Fluorescence Intensity (MFI) of Th-POK expression in iNKT cells as evaluated in **(G)**. Each dot represents a mouse. WT n=7 (in black), *Tet2/3* DKO n=7 (in purple). For each genotype male and female mice were analyzed with comparable findings. 5 independent experiments were performed. **** (p < 0.001), unpaired t test. Horizontal lines indicate the mean (s.e.m.).

We and others have previously reported that 5hmC is highly enriched in thymic CD4 SP cells (8, 46). In addition, *Tet2* and *Tet3* expression is increased as DP cells are positively selected to give rise to CD4 SP and CD8 SP cells (8) (source: Immunological Genome Project, ImmGen, **Figure 1A**). In addition, we utilized publicly available datasets (27) to investigate the expression of *Tet* genes during the process of CD4 cell lineage maturation. We discovered that *Tet1*, *Tet2* and *Tet3* become upregulated as CD4 immature SP cells become mature CD4 SP cells (**Supplementary Figure 1A**). In particular, *Tet3* shows the biggest increase (**Supplementary Figure 1A**). Interestingly, the lineage specifying transcription factor Th-POK binds to all three genes *Tet1*, *Tet2* and *Tet3* as revealed by chromatin immunoprecipitation followed by sequencing (ChIP-seq) for ThPOK in CD4 SP cells (**Supplementary Figures 1B–D**), ChIP-seq data were used from the study by (27). Specifically, Th-POK binds in the gene body of *Tet1*, the promoter of *Tet2* and is recruited in the promoter and intragenically in *Tet3* (**Supplementary Figures 1B–D**). Collectively, these findings suggest that TET proteins play a role in regulating gene expression during CD4 SP cell maturation.

We set out to discover the impact of loss of TET2 and TET3 on the gene expression program of CD4 SP cells (16). To this end, we performed low input gene expression analysis (SMART-seq) (63) as previously described (8) in highly pure, sorted CD4 SP cells (**Supplementary Figure 2**). Our comparative analysis confirmed that indeed the control, wild type CD4 SP cells were more similar to each other compared to *Tet2/3* DKO CD4 SP cells (**Supplementary Figure 3**). We discovered 816 differentially expressed genes (DEG) when comparing *Tet2/3* DKO CD4 SP cells to their wild type counterparts (**Figure 1B**). Among these 816 DEG, 255 were downregulated in *Tet2/3* DKO CD4 SP cells, while the majority (561) where upregulated in *Tet2/3* DKO CD4 SP cells (**Figure 1B**). This unbiased analysis identified that genes encoding lineage specifying factors were differentially expressed in *Tet2/3* DKO CD4 SP cells.. For instance, *Rorc* and *Tbx21*, that encode for RAR-related orphan nuclear receptor RORγt and T-bet, respectively, were among the upregulated genes in *Tet2/3* DKO CD4 SP cells. *Zbtb7b*, the gene that encodes for the lineage specifying transcription factor Th-POK, was among the downregulated genes (**Figure 1B**). It has been previously reported that deletion of Th-POK can result in redirection of CD4 SP cells to CD8 SP cells (24). However, we did not observe an increase of *Runx3* gene expression in *Tet2/3* DKO CD4 SP cells in our bulk RNA-seq data. Moreover, we did not detect a decrease of *Tet2/3* DKO CD4 SP cells (8), that would suggest that CD4 cells are redirected to CD8 SP cells. Instead, the increase of *Tet2/3* DKO CD8 SP cells that we have previously demonstrated (8) is attributed to the increase of iNKT cells that results in the emergence of innate-like CD8 cells (8). Interestingly, among the upregulated genes in *Tet2/3* DKO CD4 SP cells was *Eomes* (**Figure 1B**), as well as *Gzma* which encodes granzyme a. Notably, we also observed upregulation of genes that are normally expressed in precursor stages such as *Myb*, *Lmo2* and *Lmo4* (**Figure 1B**). These findings suggest that TET2 and TET3 regulate gene expression in the CD4 SP cells.

### Identifying conserved roles for TET proteins in regulating gene expression among conventional and unconventional T cells

Thymic iNKT cells express key lineage specifying factors which are also expressed by CD4 cells. We have previously identified that TET proteins regulate iNKT cell lineage specification (8). Thus, we interrogated whether there is a shared signature among iNKT cells and CD4 SP cells that could reveal common target genes regulated by TET proteins in conventional and unconventional T cells. To this end, we compared 1195 DEG in *Tet2/3* DKO iNKT cells (from our previous study (8)) and 889 DEG in *Tet2/3* DKO CD4 SP cells (identified in the present study) and we discovered 254 commonly DEG among the compared groups (**Figure 1C**). Among the downregulated genes, we identified *Cd4*, *Zbtb7b* and *Satb1* (**Figure 1D**). We also found that among the shared upregulated genes was *Rorc*, which encodes the lineage specifying factor RORγt. In addition, we identified genes that encode proteins involved in T cell receptor (TCR) signaling such as *Lag3*, *Nr4a2*, *Themis* and the gene *Jag1*, that encodes the Notch ligand JAG1.

### TET proteins regulate Th-POK expression in CD4 SP and iNKT cells

5hmC is highly enriched in the gene body of *Zbtb7b*, which encodes the lineage specifying transcription factor Th-POK, specifically in the CD4 SP cells when the expression of this factor is initiated (16). To evaluate if loss of TET proteins and 5hmC could impact Th-POK protein expression, we assessed Th-POK protein levels by flow cytometry in CD4 SP cells isolated from control or *Tet2/3* DKO mice. Our analysis demonstrated that Th-POK expression, evaluated by median fluorescence intensity, was reproducibly and significantly decreased in CD4 SP cells that lack concomitantly TET2 and TET3 (**Figure 1E, F**). Collectively, our data suggest that both TET2 and TET3 exert an instrumental role in regulating the magnitude of Th-POK expression in thymic CD4 SP cells. We have previously identified that *Zbtb7b* expression is significantly reduced in thymic iNKTs (8). Thus, we evaluated by flow cytometry Th-POK protein levels in total iNKT cells (**Figures 1G, H**). Our analysis reveals that *Tet2/3* DKO iNKT cells also show reduced expression of Th-POK (**Figures 1G, H**). Thus, TET2 and TET3 regulate Th-POK expression in both CD4 SP and iNKT cells.

### TET proteins control DNA demethylation across *Zbtb7b* locus in CD4 SP and iNKT cells

We then asked if loss of TET proteins would impact the cytosine methylation levels across the *Zbtb7b* locus in CD4 SP cells. To address this, we performed clone adapted template capture hybridization sequencing (CATCH-seq) (64) coupled with bisulfite sequencing to interrogate cytosine methylation status across the *Zbtb7b* locus in CD4 SP cells. We extended our analysis to include a region approximately 3.0 kb upstream of exon 1a that contains the *Zbtb7b* silencer (65) and the upstream enhancer (66), located adjacent to and upstream of the silencer. This distal enhancer regulates the initiation of *Zbtb7b* expression. Deletion of this distal enhancer results in redirection of MHC class II positively selected cells towards the CD8 lineage (62, 65). In addition, there is a proximal enhancer, located around 3.6 kb downstream of exon 1a of *Zbtb7b* (66), that is not required for initiating the expression of Th-POK after positive selection, but is instrumental in the maintenance of expression of Th-POK (65, 67).

**Figure 2:**
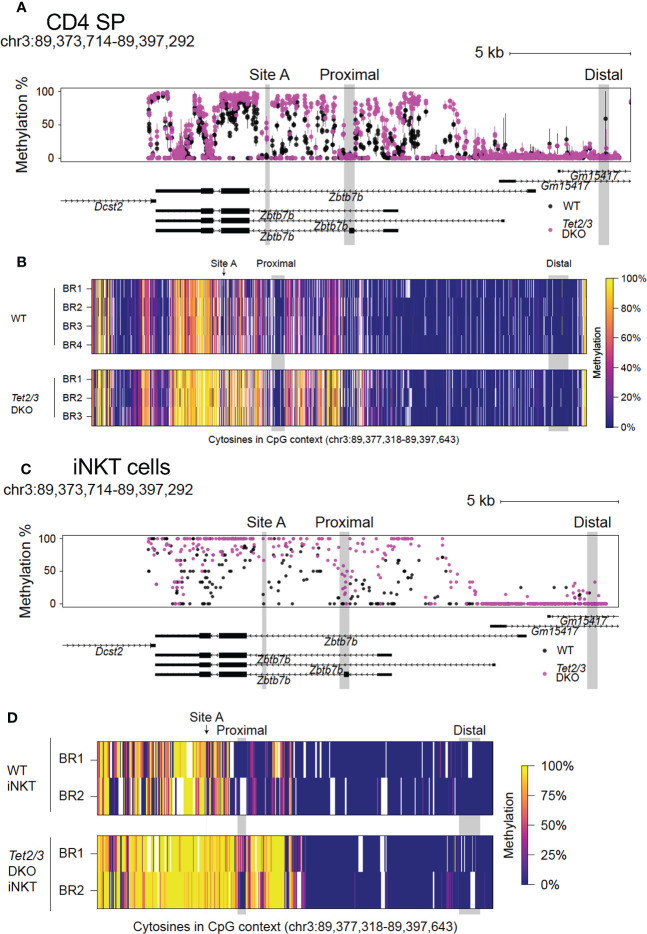
Conserved role of TET2 and TET3 in regulating DNA demethylation across the *Zbtb7b* locus in CD4 SP and iNKT cells in the thymus. **(A)** Evaluating the methylation status of cytosines in CpG context as determined by bisulfite conversion and CATCH-seq at the *Zbtb7b* locus (mouse reference genome mm10, chromosome 3: 89,373,714-89,397,292) in CD4 SP cells isolated from control (*wild type*) cells (in black) and *Tet2/3* DKO mice (in magenta). The points show means and the lines show the minimums and maximums. Site A where GATA3 is binding is highlighted in gray. Note the gain of cytosine methylation in *Tet2/3* DKO across *Zbtb7b* locus, including at site **(A)**. DNA isolated from n=4 control (shown in black) and n=3 *Tet2/3* DKO CD4 SP (depicted in magenta) biological replicates was evaluated. **(B)** Heatmap depicting the methylation status of cytosines in CpG context in the *Zbtb7b* locus and the upstream genomic region including *Zbtb7b* silencer and distal enhancer (reference genome mm10, chromosome 3: 89,373,714-89,397,292) evaluated by CATCH-seq of bisulfite treated DNA from control (wild type) (n=4 biological replicates) and *Tet2/3* DKO (n=3) CD4 SP cells. Each individual biological replicate is depicted as BR. The scale for evaluating methylation enrichment is shown. **(C)** Methylation status of cytosines in CpG context evaluated by bisulfite conversion followed by whole genome bisulfite sequencing [WGBS, data obtained from (8)] at the *Zbtb7b* locus (mouse reference genome mm10, chromosome 3: 89,373,714-89,397,292) in iNKT cells isolated from control (*wild type*) cells (in black) and *Tet2/3* DKO mice (in magenta). **(D)** Heatmap depicting the methylation status of cytosines in CpG context in the *Zbtb7b* locus and the upstream genomic region including *Zbtb7b* silencer and distal enhancer (reference genome mm10, chromosome 3: 89,373,714-89,397,292) evaluated by WGBS of bisulfite treated DNA from control (wild type, shown in black) (n=2 biological replicates) and *Tet2/3* DKO (shown in magenta) (n=2 biological replicates) iNKT cells. Each individual biological replicate is depicted as BR. The data for this analysis were obtained from (8). The scale for evaluating methylation enrichment is shown. The distal enhancer, the proximal enhancer and the site A are indicated.

Comparison of CpG methylation in control and *Tet2/3* DKO CD4 SP cells revealed a gain of intragenic methylation across the *Zbtb7b* locus upon impaired TET expression (**Figures 2A, B**). A summary of the data is depicted in **Figure 2A**. All 4 biological replicates from the control (*wild type*) mice and the 3 biological replicates from *Tet2/3* DKO mice are indicated (**Figure 2B**). We did not observe increased cytosine methylation upstream of exon 1a, in the silencer and the upstream enhancer (**Figures 2A, B**), consistent with the fact that Th-POK is expressed in *Tet2/3* DKO CD4 SP cells. Strikingly, we observed a gain of methylation in various CpGs downstream of exon 1b (**Figures 2A, B**), surrounding the proximal enhancer, that may compromise the optimal activation of the proximal enhancer. Suboptimal activation of the proximal enhancer may impact expression of Th-POK and result in CD4 SP cells that cannot sustain high levels of Th-POK, consistent with the reduced protein expression of Th-POK in *Tet2/3* DKO CD4 SP cells (depicted in **Figures 1E, F**).

In addition, we evaluated the methylation status at site A, which is located in the gene body of *Zbtb7b* gene and which has been previously reported to be a target for GATA3 (24). Our analysis revealed increased methylation of cytosines in the CpG context at this site in the *Tet2/3* DKO CD4 SP cells compared to the wild type CD4 SP cells that were devoid of methylation in this site (**Figures 2A**).

To evaluate whether TET2 and TET3 exert a conserved role in regulating Th-POK expression by regulating DNA demethylation across other lineages, we used our published whole genome bisulfite sequencing (WGBS) datasets (8), that assessed DNA methylation at single nucleotide resolution across the genome of wild type and *Tet2/3* DKO sorted iNKT cells (**Figures 2C, D**). Our analysis demonstrated a similar gain of cytosine methylation in *Tet2/3* DKO iNKT cells (**Figures 2C, D**) compared to *Tet2/3* DKO CD4 SP cells (**Figures 2A, B**). Specifically, we identified gain of methylation surrounding the proximal enhancer and site A.

### Gain of intragenic methylation and decreased binding of GATA3 at the *Zbtb7b* locus

**Figure 3:**
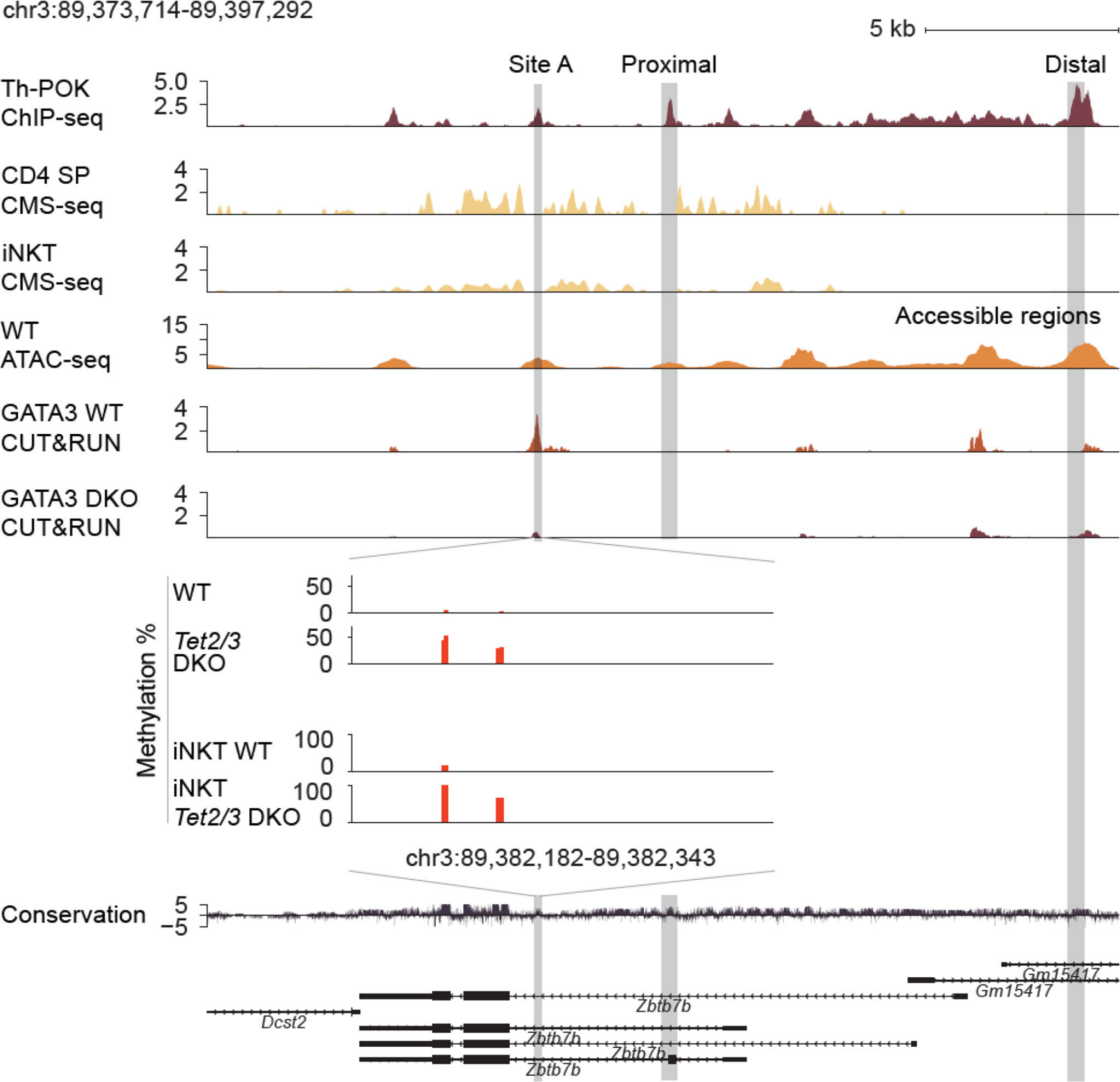
Reduced binding of GATA3 and gain of methylation across the *Zbtb7b* locus in the absence of TET2 and TET3. Genome browser snapshot of transcriptional and epigenetic regulation (evaluated by chromatin accessibility, hydroxymethylation and methylation status) of the *Zbtb7b* locus. Th-POK binding [evaluated by Th-POK ChIP-seq, data obtained from (27)], 5hmC distribution [evaluated by CMS-IP seq, (16)] in the *Zbtb7b* locus in control CD4 SP cells. GATA3 binding (evaluated by CUT&RUN, present study) is assessed in control (n=3 biological replicates) and *Tet2/3* DKO (n=2 biological replicates) CD4 SP cells. Site A is a previously identified GATA3 binding site (evaluated by ChIP) (65) that overlaps with one of the identified peaks by CUT&RUN (present study) in WT CD4 SP cells. The binding of GATA3 in site A is significantly reduced in *Tet2/3* DKO CD4 SP cells. Zoom in site A and evaluation of the methylation status by CATCH-seq. Higher level of methylation as indicated by red bars is observed in *Tet2/3* DKO CD4 SP. The arrows indicate the direction of transcription. Similar gain of methylation at site A is shown in *Tet2/3* DKO iNKT cells compared to wild type iNKT cells that are devoid of cytosine methylation in CpG context at site A. The distal enhancer, the proximal enhancer and the site A are indicated.

5hmC enrichment, which was assessed by cytosine-5-methylenesulfonate immunoprecipitation followed by sequencing (CMS-seq) (16), partially overlaps with chromatin accessible regions, identified by mining publicly available datasets of assay for transposase accessible chromatin with high throughput sequencing (ATAC-seq) (68) datasets (27), across the *Zbtb7b* gene body (**Figure 3**). Expression of *Zbtb7b* is further regulated by binding of Th-POK across the *Zbtb7b* gene body and the *Zbtb7b* silencer, as assessed by publicly available ChIP-seq datasets (27) in a feedback, autoregulatory loop as previously described (69). Th-POK binding did not appear to overlap with 5hmC enrichment, (**Figure 3**). An additional regulatory mechanism of Th-POK expression by TET proteins is to impact the binding of transcription factors that determine Th-POK expression by modulating the CpG methylation status (70). It has been reported that cytosine modification status can affect the binding of transcription factors across the DNA (70). For instance, the vast majority of transcription factors cannot bind to methylated cytosines, however pioneer transcription factors have been reported to recognize and bind to 5mC. In addition, oxi-mCs can be specifically recognized by transcription factors (4) and thus their absence can inhibit transcription factor binding. GATA3 is known to induce Th-POK expression in CD4 SP cells (24) and is necessary for CD4 lineage commitment. GATA3 is known to bind to the *Zbtb7b* locus upstream of exon 2, in chromatin accessible region, at site A (24) (**Figure 3**). Interestingly, in this region we discover a gain of CpG methylation upon *Tet2/3* deletion. Thus, we tested GATA3 binding in control and *Tet2/3* DKO CD4 SP cells using Cleavage Under Targets and Release Using Nuclease (CUT&RUN) (71). Our data revealed that GATA3 binds strongly to the *Zbtb7b* locus in wild type CD4 SP cells (3 biological replicates were evaluated), however there is a significant reduction of GATA3 binding in *Tet2/3* DKO CD4 SP cells (2 biological replicates were used) (**Figure 3**). These data suggest that GATA3 binds accessible intragenic regions in control CD4 SP cells (**Figure 3**). Gain of methylation upon TET2/3 loss presumably compromise the efficiency of GATA3 binding in the *Zbtb7b* locus and might contribute to the subsequent reduced expression of Th-POK in CD4 SP cells (**Figure 3**, **Figures 1E, F**).

Notably, we have previously reported that 5hmC is significantly enriched across the gene body of GATA3 in thymocytes as well as in Th2 cells (16). Similar detailed findings have been recently reported regarding 5hmC enrichment across *Gata3* during *in vitro* Th2 polarization (72). *Gata3* mRNA expression was not significantly reduced in *Tet2/3* DKO CD4 SP cells, however there was a clear trend for reduced expression (**Supplementary Figure 4A**). We then examined protein expression levels of GATA3 in CD4 SP cells from control and *Tet2/3* DKO mice by flow cytometry. This analysis confirmed a reproducible and significant trend of reduced expression of GATA3 in *Tet2/3* DKO CD4 SP cells (**Supplementary Figures 4B, C**). Thus, the significant reduction of GATA3 binding at site A that we identify in *Tet2/3* DKO CD4 SP cells could be the cumulative effect of increased methylation at the site and reduced expression of GATA3.

### TET2 that lacks enzymatic activity fails to restore iNKT cell expansion in the thymus in the concomitant absence of TET3

**Figure 4:**
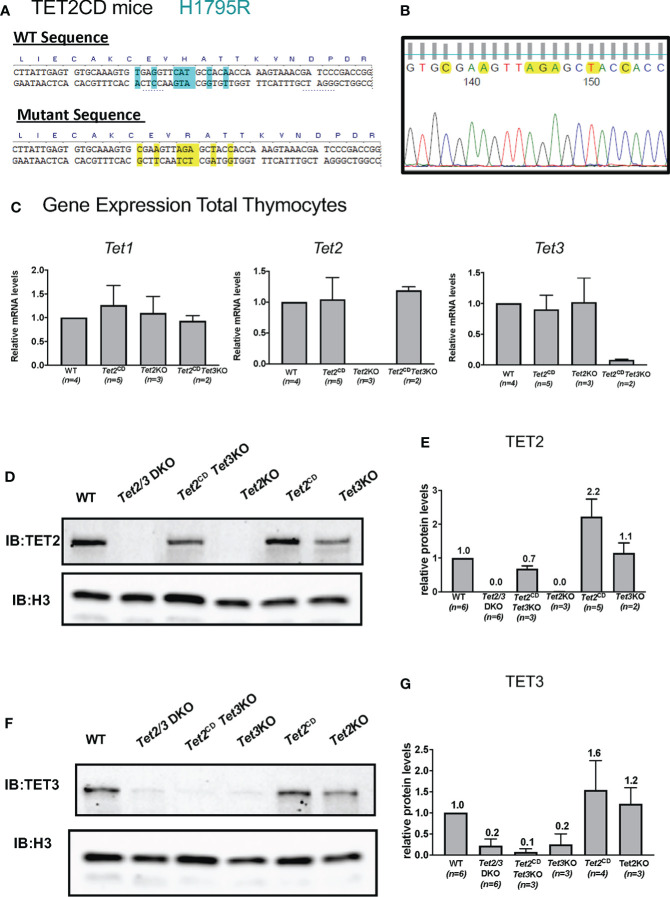
Characterization of *Tet2* Catalytic Dead (*Tet*2^CD^) *Tet2^CD^ Tet3^flx/flx^ CD4Cre* (*Tet2^CD^ Tet3* KO) mice. **(A)** Depicting the point mutations that result in H1795R catalytic dead TET2 mice. The substitution of histidine (H) with arginine (R) compromises the ability to bind Fe2+ that is critical for the catalytic activity of TET2. **(B)** Sanger sequencing of genomic DNA indicating the mutations at the TET2 catalytic domain. **(C)** Gene expression evaluation by real time PCR for *Tet1*, *Tet2* and *Tet3* in total thymocytes isolated by control (WT), *Tet2* ^CD^ *Tet3* KO, *Tet2* ^CD^ and *Tet2* KO. Data from one representative experiment are shown. The total number of evaluated mice is indicated. **(D)** Western blot depicting TET2 expression in nuclear extracts from total thymocytes isolated from control (WT), *Tet2/3* DKO, *Tet2* ^CD^ *Tet3* KO, *Tet2* KO, *Tet2* ^CD^ and *Tet3* KO. Histone 3 (H3) was used as loading control. Data from one representative experiment are shown. **(E)** Quantification of western blots showing expression of TET2. The number of mice evaluated for each genotype is shown in parenthesis. Control (WT) n=6, *Tet2/3* DKO n=6, *Tet2* ^CD^ *Tet3* KO n=3, *Tet2* KO n=3, *Tet2* ^CD^ n=2 and *Tet3* KO n=2. **(F)** Western blot depicting TET3 expression in nuclear extracts from total thymocytes isolated from control (WT), *Tet2/3* DKO, *Tet2* ^CD^ *Tet3* KO, *Tet3* KO, *Tet2* ^CD^ and *Tet2* KO. Histone 3 (H3) was used as loading control. **(G)** Quantification of western blots showing expression of TET3. The number of mice evaluated for each genotype is shown in parenthesis. control (WT) n=6, *Tet2/3* DKO n=6, *Tet2* ^CD^ *Tet3* KO n=3, *Tet3* KO n=3, *Tet2* ^CD^ n=4 and *Tet2* KO n=3.

Notably, TET2 has been suggested to exert additional, non-catalytic roles in regulating gene expression in hematopoietic populations (48, 49, 73) and potentially in controlling the proliferation of iNKTs (8). To assess if TET2 might play a catalytic independent role in regulating conventional and unconventional T cell lineage specification, we used *Tet2* ^CD^ mice (**Figure 4A**) that express full length TET2 protein with point mutations, which compromise iron binding and thus render TET2 catalytically inactive (51). We confirmed the point mutations by Sanger sequencing (**Figure 4B**). In addition, we confirmed that thymocytes isolated from the *Tet2* ^CD^ express TET2 both at the RNA level by quantitative PCR (**Figure 4C**) and at the protein level (**Figures 4D, E**, original western blots available in **Supplementary Figure 5A**). Thymic development was not affected in *Tet2* ^CD^ mice (**Supplementary Figure 6**), suggesting that TET2 full length protein that is catalytically inactive does not exert a dominant negative effect by preventing recruitment of wild type TET3 to its target genes.

To investigate whether TET2 catalytically inactive protein has shared redundant functions with TET3 in regulating thymic T cell and iNKT cell development, we generated *Tet2* ^CD^ *Tet3* ^flx/flx^ CD4Cre (*Tet2* ^CD^ *Tet3* KO) mice. The goal was to dissect whether TET2 could exert non-catalytic roles in regulating Th-POK expression in collaboration with TET3. Our analysis showed that *Tet2* ^CD^ *Tet3* KO mice developed similar, aggressive phenotype with *Tet2/3* DKO mice and exhibited splenomegaly and lymphadenopathy. Thus, we focused our analysis in young mice (21-28 days old) that appeared normal. First, we evaluated the gene expression of *Tet1*, *Tet2* and *Tet3* by real time PCR using RNA isolated from wild type, *Tet2* ^CD^, *Tet2* KO and *Tet2* ^CD^ *Tet3* KO total thymocytes. While *Tet2* ^CD^ *Tet3* KO thymocytes do not express *Tet3*, they do express *Tet1* and *Tet2* (**Figure 4C**).

Next, we assessed the protein expression levels of TET2 and TET3. Our data confirm that thymocytes isolated from *Tet2* ^CD^ *Tet3* KO mice express TET2 but do not express TET3. In addition, we included as controls thymocytes isolated from wild type, *Tet*2/3 DKO mice, *Tet2* KO, *Tet2* ^CD^ and *Tet3* KO mice (**Figures 4D–G**, original, unprocessed western blots available in **Supplementary Figure 5A**). Moreover, as CD4 SP cells express higher levels of TET2 compared to DP cells [**Figure 1A** and (8, 46)], we evaluated TET2 protein expression levels specifically at CD4 SP cells that were isolated by FACS sorting from control and *Tet2* ^CD^ *Tet3* KO mice. Our analysis confirmed that CD4 SP cells isolated from *Tet2* ^CD^ *Tet3* KO mice express TET2 protein at comparable levels to wild type CD4 SP cells (**Supplementary Figure 7**, original western blots available in **Supplementary Figure 5B**).

### Enzymatic activity of TET2 is required for iNKT cell lineage expansion and specification

**Figure 5:**
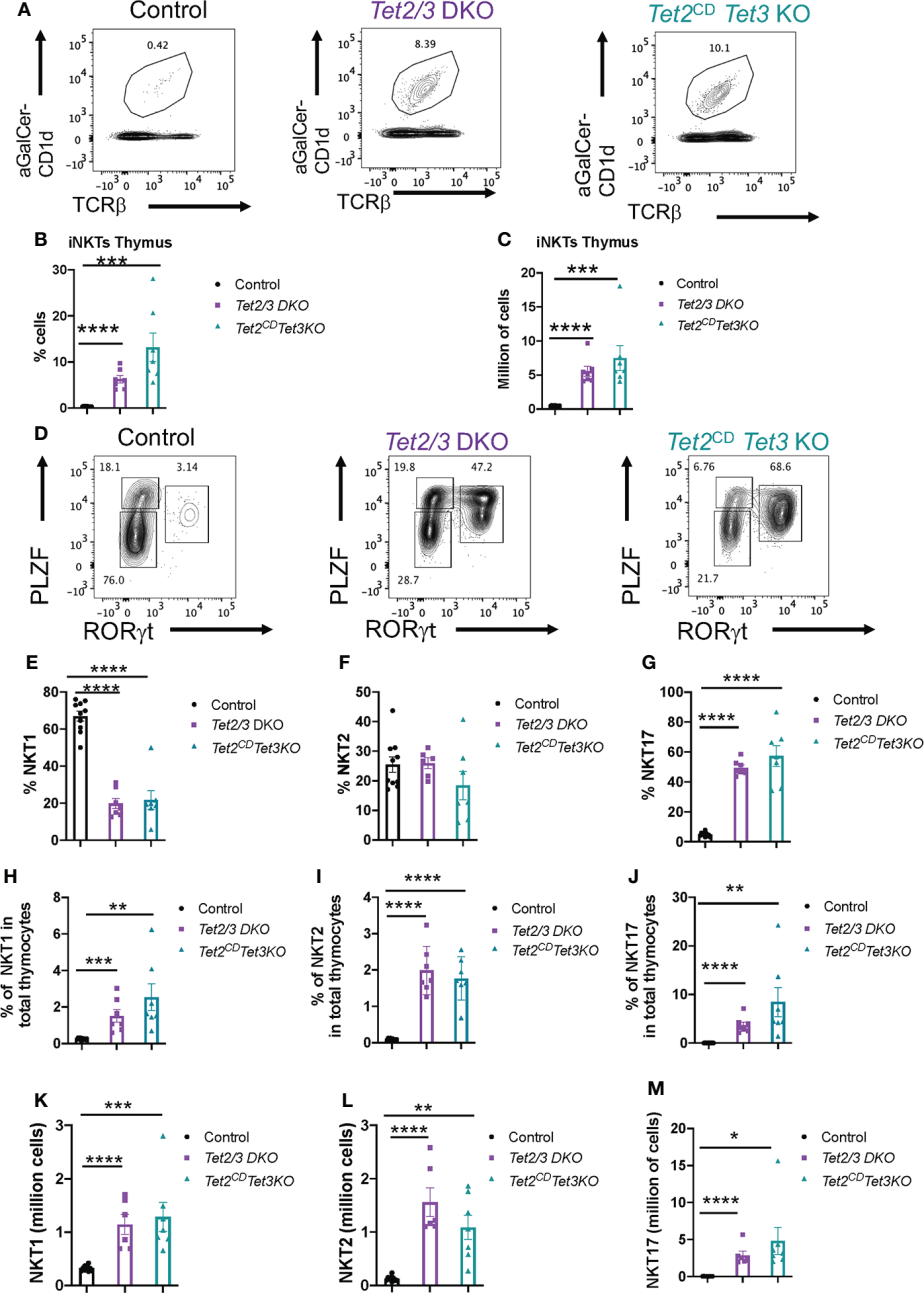
*Tet2* ^CD^ *Tet3* KO iNKT cells show expansion and NKT17 cell lineage skewing. **(A)** Representative flow cytometry plots evaluating aGalactosylseramide (aGalCer) loaded CD1d tetramer binding and TCRβ expression in thymocytes isolated from control, *Tet2/3* DKO and *Tet2* ^CD^ *Tet3* KO mice. The aGalCer-CD1d tetramer+ TCRβ+ cells are iNKT cells. **(B)** Percentage of iNKT cells (identified as described in **(A)** in the thymus isolated from control (in black), *Tet2/3* DKO (in purple), *Tet2* ^CD^ *Tet3* KO (in cyan) mice. **(C)** Number of iNKT cells in the thymus of control (in black), *Tet2/3* DKO (in purple), *Tet2* ^CD^ *Tet3* KO (in cyan) mice. **(D)** Representative flow cytometry plots evaluating the expression of transcription factors PLZF and RORγt in thymus isolated from control, *Tet2/3* DKO and *Tet2* ^CD^ *Tet3* KO mice. PLZF^low^ RORγt ^negative^ cells are NKT1, PLZF ^high^ RORγt ^negative^ are NKT2 and PLZF ^intermediate^ RORγt ^positive^ cells are NKT17 cells. **(E)** Percentage of NKT1 cells, **(F)** NKT2 and **(G)** NKT17 cells among total iNKT cells in the thymus isolated from control (in black), *Tet2/3* DKO (in purple), *Tet2* ^CD^ *Tet3* KO (in cyan) mice. **(H)** Percentage of NKT1 cells, **(I)** NKT2 cells and **(J)** NKT17 cells among total thymocytes isolated from control (in black), *Tet2/3* DKO (in purple), *Tet2* ^CD^ *Tet3* KO (in cyan) mice. **(K)** Number of NKT1, **(L)** NKT2 and **(M)** NKT17 cells in the thymus isolated from control (in black), *Tet2/3* DKO (in purple), *Tet2* ^CD^ *Tet3* KO (in cyan) mice. For B, C, E-M: Each dot represents a mouse. 5 independent experiments were performed. Male and female mice for each genotype were analyzed. Control n=10, *Tet2/3* DKO n=6, *Tet2* ^CD^ *Tet3* KO n=7 **** (p < 0.0001),*** (p < 0.001), ** (p < 0.01), * (p < 0.05) unpaired t test. Unless otherwise indicated the difference is not statistically significant. Horizontal lines indicate the mean (s.e.m.).

We have previously established a fundamental role for both TET2 and TET3, working in a cooperative fashion, in regulating iNKT cell expansion and lineage specification (8). However, when we investigated DNA methylation in *Tet2/3* DKO iNKT cells we discovered a limited number of differentially methylated regions revealing a focal role of TET proteins in regulating DNA demethylation (8). *Tet3* KO mice showed iNKT cell lineage skewing but the frequency and numbers of iNKT cells were normal in these mice (8). However, abundancy and lineage specification of iNKT cells was normal in *Tet2* KO mice (8). Interestingly, it has been reported that TET2 exerts catalytic independent roles to regulate lymphoid proliferation (48). Thus, an open question was if TET2 could exert additional, catalytic independent roles to regulate iNKT cell proliferation. We isolated thymocytes from control, *Tet2/3* DKO and *Tet2* ^CD^ *Tet3* KO mice and performed staining with a tetramer of the MHC class I CD1d that is loaded with a-Galactosylceramide (aGal-Cer), a lipid that iNKT cells can recognize and bind, and TCRβ. Cells that were positive for aGalCer-CD1d and expressed intermediate levels of TCRβ were identified as iNKT cells (**Figure 5A**). Our analysis revealed that iNKT cells exhibit a dramatic expansion in *Tet2* ^CD^ *Tet3* KO iNKT mice, similar to the *Tet2/3* DKO iNKT cell expansion that we have previously reported (8) (**Figure 5A**). This increase of the percentage of iNKT cells in the thymus was reproducible and significant (**Figure 5B**) and it was also observed at the level of numbers of cells (**Figure 5C**).

Next, we asked whether iNKT cell lineage specification was affected in the thymus of *Tet2* ^CD^ *Tet3* KO mice in comparison to wild type and *Tet2/3* DKO mice. To this end we performed a flow cytometry experiment to investigate the expression of key lineage specifying transcription factors in iNKT cells (**Figure 5D**). Specifically, we focused on the protein expression levels of the iNKT cell lineage specifying factor PLZF and the NKT17 cell lineage specifying factor RORγt. Our analysis confirmed that in thymic iNKT cells isolated from wild type mice the vast majority of the cells expressed low levels of PLZF and they did not express RORγt (**Figure 5D**), consistent with the prevalence of NKT1 subset in the thymus of C57/BL6 mice. However, in sharp contrast to the control iNKTs, the *Tet2* ^CD^ *Tet3* KO iNKT cells exhibit a striking upregulation of the NKT17 subset that is characterized by RORγt expression and intermediate levels of PLZF expression (**Figure 5D**). The NKT17 subset in the thymus isolated from control mice is the least frequent (**Figure 5D**). We also investigated the representation of the PLZF high, RORγt negative subset that represents NKT2 cells (**Figure 5D**). Overall, our analysis revealed that *Tet2* ^CD^ *Tet3* KO iNKT subsets were comparable in percentage and numbers to the *Tet2/3* DKO iNKT cells (**Figures 5D–M**). The experimental results showed a significant increase in frequency of NKT17 cells and a decrease in the frequency of NKT1 cells compared to control mice (**Figures 5D–G**). Notably, the frequency of iNKT cells in the thymus was strikingly higher in the mutant mice compared to the control. Thus, we proceeded to analyze the frequency of iNKT subsets among total thymocytes (**Figures 5H–J**). This approach reveals that the NKT2 population is significantly upregulated in both *Tet2/3* DKO and *Tet2* ^CD^ *Tet3* KO mice compared to control mice (**Figures 5H–J**). In addition, the mutant mice exhibited a significant increase in absolute numbers of all the three iNKT subsets (NKT1, NKT2 and NKT17) (**Figures 5K–M**). Collectively, our results establish an instrumental role of the enzymatic activity of TET2 that, in cooperation with TET3, regulates iNKT cell expansion and differentiation.

### Development of innate like CD8 cells in the thymus of *Tet2* ^CD^ *Tet3* KO mice

**Figure 6:**
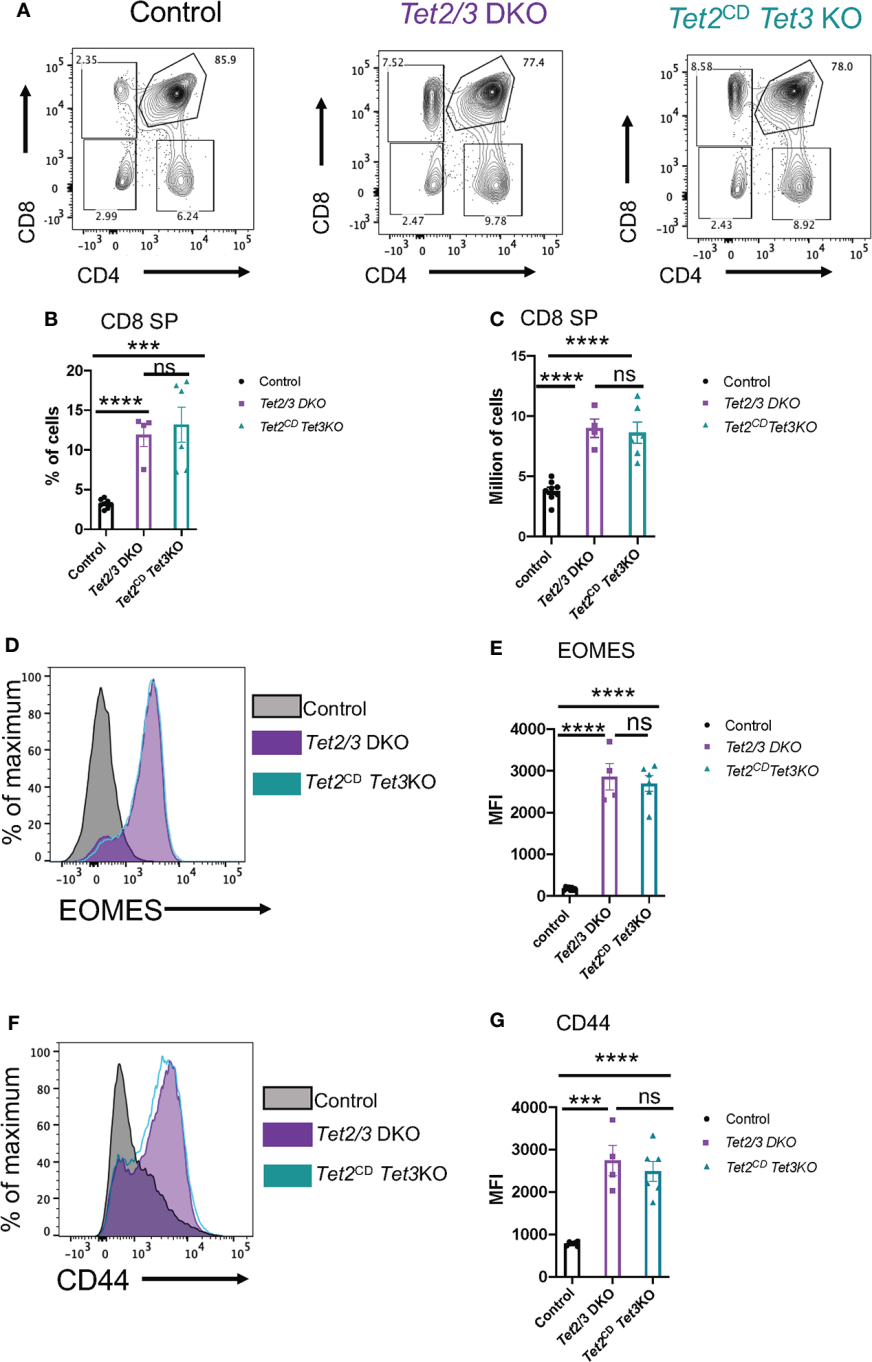
*Tet2* ^CD^ *Tet3* KO CD8 SP cells exhibit innate like characteristics. **(A)** Representative flow cytometry plots evaluating CD4 and CD8 expression in the surface of thymocytes (excluding iNKT cells) isolated from control (wild type), *Tet2/3* DKO and *Tet2* ^CD^ *Tet3* KO mice. **(B)** Percentage of CD8 SP cells (determined as described in A) in the thymus isolated from control (in black), *Tet2/3* DKO (in purple), *Tet2* ^CD^ *Tet3* KO (in cyan) mice. **(C)** Number of CD8 SP cells in the thymus of control (in black), *Tet2/3* DKO (in purple), *Tet2* ^CD^ *Tet3* KO (in cyan) mice. **(D)** Representative histograms evaluating the expression of the transcription factor EOMES in CD8 SP cells. Gray is indicating control cells, purple *Tet2/3* DKO and cyan *Tet2* ^CD^ *Tet3* KO. **(E)** Median fluorescence intensity (MFI) of EOMES expression in control (in black), *Tet2/3* DKO (in purple) and *Tet2* ^CD^ *Tet3* KO (in cyan) CD8 SP cells. **(F)** Representative histograms evaluating the expression of CD44 in CD8 SP cells. Gray is indicating control cells, purple *Tet2/3* DKO and cyan *Tet2* ^CD^ *Tet3* KO. **(G)** Median fluorescence intensity (MFI) of CD44 expression in control (in black), *Tet2/3* DKO (in purple) and *Tet2* ^CD^ *Tet3* KO (in cyan) CD8 SP cells. For **(B, C, E, G)**: Each dot represents a mouse. 4 independent experiments were performed. Male and female mice for each genotype were analyzed. Control n=8, *Tet2/3* DKO n = 4, *Tet2* ^CD^ *Tet3* KO n = 6 **** (p < 0.0001),*** (p < 0.001), ns, non-significant, unpaired t test. Horizontal lines indicate the mean (s.e.m.).

We further evaluated thymic development in *Tet2* ^CD^ *Tet3* KO mice. Increased numbers of iNKT cells and secretion of cytokine IL-4 results in the emergence of a thymic population of unconventional CD8 SP cells with memory characteristics (74). These innate like CD8 SP cells upregulate CD44 and express the T-box transcription factor Eomesodermin (EOMES) (75, 76). We observed an increase in the percentage of CD8 SP cells in the thymus of *Tet2* ^CD^ *Tet3* KO mice, that was reminiscent of the increased CD8 SP representation in the thymus of *Tet2/3* DKO mice (**Figures 6A, B**). This increase was reproducible and significant both in terms of frequency (evaluated as percentage of cells) and in absolute numbers (**Figures 6A–C**). To assess if the expanded *Tet2* ^CD^ *Tet3* KO CD8 SP cells had features of activated, innate like cells (8) we evaluated by flow cytometry the expression of the transcription factor EOMES. Our data confirmed that *Tet2* ^CD^ *Tet3* KO CD8 SP cells upregulate EOMES at comparable levels to *Tet2/3* DKO CD8 SP cells (**Figures 6D, E**). On the other hand, control CD8 SP cells did not express EOMES (**Figures 6D, E**). Similarly, *Tet2* ^CD^ T*et3* KO CD8 SP cells show increased levels of CD44 expression, an additional hallmark of activated, memory like CD8 cells (**Figures 6F, G**). Overall, as a result of iNKT cell expansion in the thymus of *Tet2* ^CD^ *Tet3* KO mice, we identify the emergence of memory CD8 SP cells consistent with the observed phenotype in *Tet2/3* DKO mice.

### TET2 catalytic activity is required for optimal Th-POK expression in CD4 SP and iNKT cells

**Figure 7:**
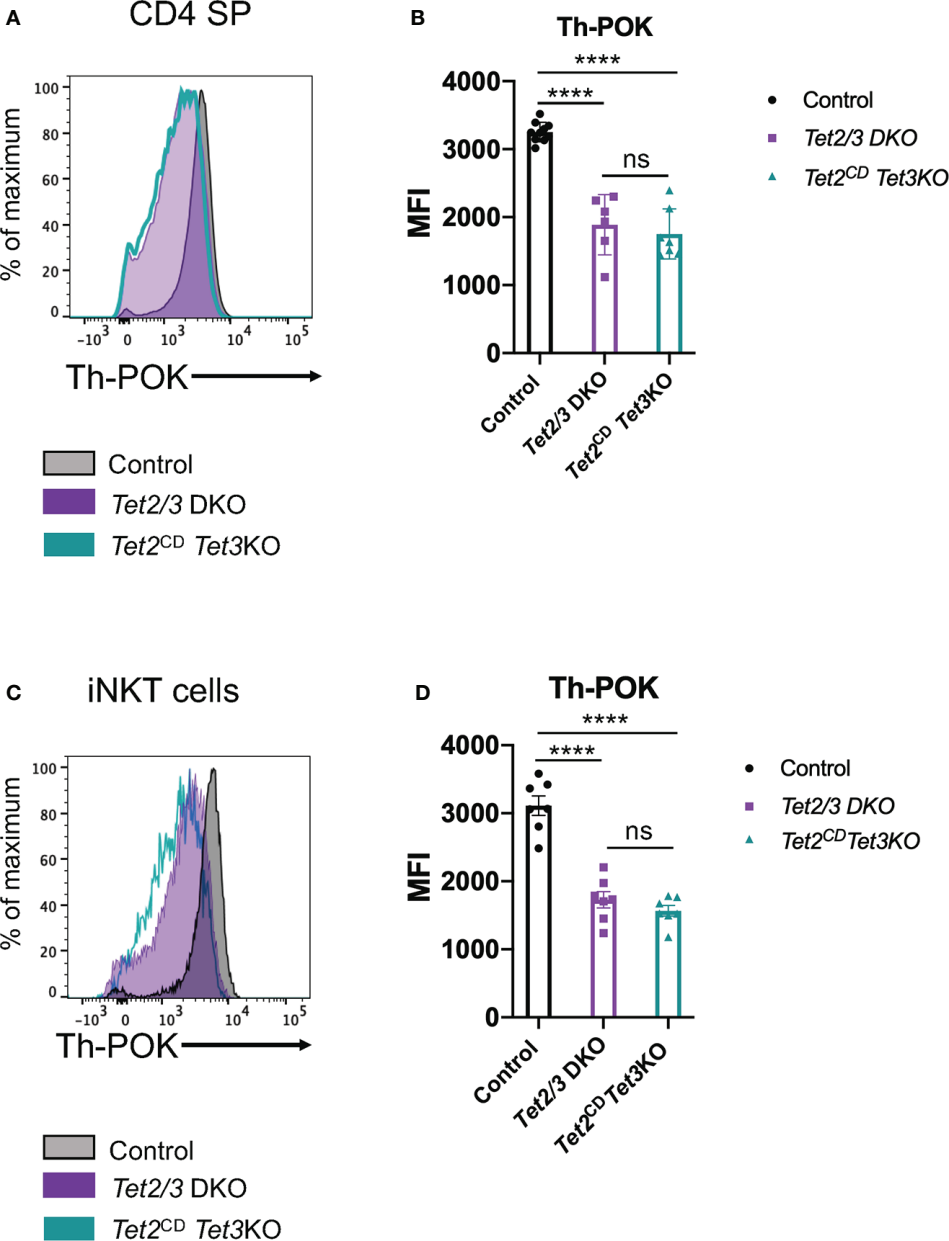
TET2 catalytic activity is required to fine tune Th-POK expression in cooperation with TET3 in CD4 SP cells and iNKT cells. **(A)** Representative histogram depicting Th-POK expression, assessed by flow cytometry, in control (black), *Tet2/3* DKO (purple), *Tet2* ^CD^ *Tet3* KO (cyan) in CD4 SP cells. **(B)** Median fluorescence intensity (MFI) of Th-POK expression in control (in black), *Tet2/3* DKO (in purple) and *Tet2* ^CD^ *Tet3* KO (in cyan) CD4 SP cells. Each dot represents a mouse. 5 independent experiments were performed. Male and female mice for each genotype were analyzed. Control n=10, *Tet2/3* DKO n=6, *Tet2* ^CD^ *Tet3* KO n=7 **** (p < 0.0001), ns: non-significant, unpaired t test. Horizontal lines indicate the mean (s.e.m.). **(C)** Representative histogram depicting Th-POK expression, assessed by flow cytometry, in control (black), *Tet2/3* DKO (purple), *Tet2* ^CD^ *Tet3* KO (cyan) in iNKT cells. **(D)** Median fluorescence intensity (MFI) of Th-POK expression in control (in black), *Tet2/3* DKO (in purple) and *Tet2* ^CD^ *Tet3* KO (in cyan) iNKT cells. Each dot represents a mouse. 5 independent experiments were performed. Male and female mice for each genotype were analyzed. Control n=7, *Tet2/3* DKO n = 7, *Tet2* ^CD^ *Tet3* KO n = 7 **** (p < 0.0001), ns, non-significant, unpaired t test. Horizontal lines indicate the mean (s.e.m.).

Next, we asked if expression of full length TET2 protein with compromised enzymatic activity could restore the expression of Th-POK in CD4 SP cells that lacked TET3. To this end, we performed a flow cytometry experiment to interrogate the expression levels of this transcription factor (**Figure 7A**). Our analysis revealed that Th-POK expression could not be restored in the presence of TET2^CD^ upon simultaneous deletion of TET3. Instead, expression levels of Th-POK were consistently reduced in both *Tet2/3* DKO and *Tet2* ^CD^ *Tet3* KO CD4 SP cells compared to CD4 SP cells isolated from thymus of wild type mice (**Figures 7A, B**). Furthermore, we confirm that Th-POK expression is significantly reduced in *Tet2/3* DKO and *Tet2* ^CD^ *Tet3* KO iNKT cells (**Figures 7C, D**). Thus, we establish a critical role for TET2 enzymatic activity that, in cooperation with TET3, regulate the magnitude of Th-POK expression in CD4 SP and iNKT cells,

## Discussion

Our data suggest a multifaceted role of TET proteins and 5hmC in thymic T cell lineage specification. In this study, we establish a critical role of TET proteins in regulating gene expression in the CD4 lineage. First, we demonstrate that TET2 and TET3 shape gene expression in CD4 SP and iNKT cells (**Figure 1**). TET proteins act to regulate DNA demethylation at the *Zbtb7b* locus (**Figure 2**) affecting the intensity of expression of the lineage specifying transcription factor Th-POK in both CD4 and iNKT cells (**Figure 1**). Our findings suggest that TET2 and TET3 are not required for the initial expression of Th-POK that is regulated through the distal enhancer. Instead, TET proteins and their enzymatic activity are instrumental for maintaining the optimal expression of Th-POK. These findings enforce the discoveries that TET proteins through their enzymatic activity set the stage for maintenance of stable gene expression of the transcription factor FOXP3, that shapes the identity of regulatory T cells (77–81). In addition, it has been reported that TET proteins can demethylate an enhancer that is required for the stable expression of CD4 during *in vitro* culture and expansion (18), as well as *in vivo* (82), providing an additional example where TET mediated DNA demethylation of a locus is critical for stable gene expression and maintenance of cell identity. Thus, an emerging concept is that TET mediated DNA demethylation might be critical for the stability and the regulation of the magnitude of gene expression along the life-span of the cells.

Notably, how TET protein expression is regulated in CD4 cells has not been explored. In the present study, we also report that *Tet* genes are targets of Th-POK in thymic CD4 SP cells (**Supplementary Figure 1**). Thus, Th-POK might play regulatory roles to control TET protein expression during the process of CD4 SP cell lineage maturation. This could be at least partially due to TET protein mediated de-methylation of *Zbtb7b* locus and the maintenance of gene expression. In addition, oxi-mCs might play a role in the recruitment of Th-POK across the genome (4). Further studies are required to elucidate the precise role of Th-POK in regulating expression of TET proteins.

Moreover, our data establish an additional layer by which TET proteins regulate gene expression in the CD4 lineage by affecting both transcription factor expression and binding across the genome. Specifically, we demonstrate that GATA3 is less expressed in *Tet2/3* DKO CD4 cells compared to control (wild type) cells. There is reduced binding of GATA3 at site A of *Zbtb7b* locus, that might contribute to the overall reduced Th-POK expression in *Tet2/3* DKO cells. Our findings suggest redundant regulatory roles for TET2 and TET3 in fine tuning Th-POK expression and GATA3 expression and potentially binding in CD4 SP cells. Notably, TET2 has been suggested to exert additional, non-catalytic roles in regulating gene expression in hematopoietic populations (48) and potentially in controlling the proliferation of iNKTs (8). Our data demonstrate that both TET2 and TET3 exert an instrumental role to regulate DNA demethylation of *Zbtb7b* to regulate the magnitude of Th-POK expression and to permit the optimal binding of GATA3 in the *Zbtb7b* locus and across the genome of CD4 SP cells.

A critical question is to understand how TET proteins are recruited at the DNA to regulate DNA demethylation of their target genes in distinct T cell subsets (11). It is well established that TET2 cannot bind to the DNA as it lacks a CXXC DNA binding domain (3). In addition, while TET3 maintains a CXXC DNA binding domain, the focal DNA demethylation that has been observed for instance in *Tet2/3* DKO iNKT cells (11) as well as in TET deficient hematopoietic stem cells (9) demonstrates a cell specific, targeted action of TET proteins. The cell specificity of TET mediated DNA demethylation can be attributed to transcription factors that interact with TET proteins and direct them to specific loci across the genome. Indeed, various interactions among TET proteins and transcription factors have been described (83), for instance during the process of B cell differentiation (15) and during reprogramming (13). In addition, inhibition of interaction of TET2 with Wilm’s tumor gene (WT1) has been reported to promote tumorigenesis (12). However, our knowledge regarding factors that can mediate recruitment of TET proteins to their target genes during thymic development remains limited. Intriguingly, GATA3 has been shown to interact with TET2 in the estrogen receptor (ER) complex through use of rapid immunoprecipitation mass spectrometry of endogenous proteins and this interaction was critical for 5hmC deposition at ER target genes (84). Thus, an additional layer of regulating GATA3-mediated transcriptional activation in CD4 cells might be through loss of TET2 (and potentially TET3) mediated DNA demethylation. In other words, GATA3 might still bind at a locus but the optimal demethylation and subsequent activation of the gene might be compromised. A similar hypothesis has been postulated for GATA3 and TET2 cooperation in Th2 cells for inducing IL-4 expression (72). Further studies are required to elucidate which are the transcription factors that mediate recruitment of TET proteins in the DNA at distinct lineages during the process of T cell lineage specification and commitment.

Notably, loss of TET proteins and their capacity to confer epigenetic regulation by DNA demethylation reduces expression or impairs the binding and subsequent function of various transcription factors. This is distinct than what has been observed upon deletion of a single, specific transcription factor that results in shutting down the gene expression network that this given factor orchestrates. For instance, loss of Th-POK blocks CD4 maturation and redirects cells to become CD8 cells. Instead, our findings showcase how epigenetic regulators, like TET proteins, fine tune gene expression, and thus impact simultaneously various fundamental transcription factors affecting broadly gene expression. Moreover, in thymic *Tet2/3* DKO CD4 SP cells we report upregulation of cytotoxic genes such as *Eomes* and *Gzma* that might be due to downregulation of Th-POK (69). A gene that was found to be downregulated both in *Tet2/3* DKO CD4 SP cells and *Tet2/3* DKO iNKT cells was *Cd4* (**Figure 1**). Importantly, we have also established an instrumental role of Th-POK downregulation in the observed lineage skewing of *Tet2/3* DKO iNKT cells (8). Specifically, we demonstrated that expressing Th-POK in *Tet2/3* DKO iNKT cells is sufficient to suppress aberrant RORγt upregulation in these cells (8). In addition, we identify upregulation of genes such as *Myb* and *Lmo2*, which characterize earlier developmental stages (**Figure 1**). Aberrant expression of precursor genes could result in hyperproliferation and ultimately malignant transformation of blood cells (11).

Indeed, TET proteins act as tumor suppressors and safeguard cells from aberrant proliferation. Specifically, TET2 has been frequently reported to be mutated in hematological malignancies (85), including angioimmunoblastic T cell lymphoma (AITL) (86–88) and peripheral T cell lymphoma, non-otherwise specified (PTCL-NOS) (89). It has been suggested that TET2 mutations are an early event during the process of malignant transformation and confer proliferative advantage to the mutant cells (85, 89). Importantly, loss of TET proteins in various innate and adaptive immune populations compromises the process of maturation and establishment of cell fate, resulting in uncontrolled proliferation (11, 90). However, the mechanisms by which TET proteins safeguard controlled proliferation and protect from the rise of hematological malignancies and inflammatory diseases remain poorly understood. While loss of catalytic activity of TET proteins is strongly associated with hematopoietic stem cell hyperproliferation and myeloid malignancies (12, 46, 91), it has been recently suggested that the enzymatic activity of TET2 might be dispensable for its tumor suppressive role at least in some lymphoid malignancies (48) as well as in the context of inflammation (49).

In our previous study regarding the *in vivo* role of TET proteins in regulating iNKT cell lineage specification and expansion we established that TET3 catalytic activity was important for the NKT17 lineage skewing and we hypothesized that TET2 might exert additional, enzymatic-independent roles to control iNKT expansion (8). In the present study, to follow up on this important question, we utilized mice that express full length TET2 that has impaired capacity to bind iron, a critical cofactor, required for enzymatic activity and cytosine oxidization (**Figure 4**). The thymic differentiation in the *Tet2* ^CD^ *Tet3* KO mice was comparable to the *Tet2/3* DKO mice. We identified iNKT cell lineage skewing and expansion (**Figure 5**) as well as emergence of innate like CD8 SP cells (**Figure 6**). We also confirmed that Th-POK expression is affected by TET2 and TET3 in a TET2 dependent catalytic manner (**Figure 7**). Collectively, our data provide a causative link between the catalytic dependent role of TET proteins and the establishment of thymic T cell lineage specification.

An emerging question is to understand what is the physiological impact of the observed changes in the lineage specification that occurs in the thymus upon concomitant deletion of TET2 and TET3. For instance, mouse models that lack specifically TET2 in T cells exhibit defects in immune response against viral infection (92). Similarly, loss of TET2 in innate lymphoid cells results in less efficient immune response against bacterial infection (93). Moreover, a critical role for TET mediated DNA demethylation has been established for the stability and the optimal suppressive function of regulatory T cells (77–81, 94, 95). Thus, to pursue further functional analysis in our system, the challenge is to overcome the multiple immunological issues related to TET2/3 loss in T cells; such as aberrant iNKT cell development and expansion (8), emergence of CD8 innate like T cells (8) and the well-established instability of the regulatory T cell lineage (77) that collectively result in an aggressive disease that causes accelerated death of the *Tet*2/3 DKO mice between 7 and 8 weeks old (8, 77). Establishing new mouse models, to delete *Tet* genes at various developmental stages and at specific T cell subsets will shed light on the precise biological roles of TET2 and TET3 in immune response and blood cancer development.

## Data availability statement

The datasets presented in this study can be found in online repositories. The names of the repository/repositories and accession number(s) can be found in the article/**Supplementary Material**. The datasets generated in this study have been deposited in the Gene Expression Omnibus (GEO) public repository: SuperSeries: GSE206450.

## Ethics statement

All the experiments conducted in this study were compliant with the ethical regulations approved by the UNC Institutional Animal Core and Use Committee (IACUC) and the protocol 20-013.

## Author contributions

TÄ analyzed all the genome-wide sequencing datasets, generated the related figures, interpreted data and wrote the genome wide data analysis methods section. DT performed CUT&RUN experiments, western blot and real-time PCR experiments, assisted in primary cell isolation and mouse colony management and analyzed data. MC, MS and YX designed and generated the *Tet2* ^CD^ mice. ASB provided the *Tet2* ^CD^ mice, critically read and commented on the manuscript. AT conceived and supervised the study, designed, performed and analyzed experiments, secured funding and wrote the manuscript with input from all the authors. All authors agree to the content of the manuscript. All authors contributed to the article and approved the submitted version.

## Funding

This work was supported by NIH grant (R35-GM138289), Supplement R35-GM138289-02S1 from National Institute of General Medicinal Sciences (NIGMS) (to AT), and UNC Lineberger Compehensive Cancer Center Startup funds (to AT) and NIH grant 5-R01CA163834 (to ASB). AT is a recipient of an IBM Chancellor’s Career Development Award. A.S.B. is the recipient of the NIH grant 5-R01CA163834.

## Acknowledgments

We wish to thank Ms. Kayla Harrison (UNC DCM Colony Management Core) for excellent mouse colony management. We also wish to acknowledge Ms. Janet Dow, Mr. Roman Bandy and Ms. Ayrianna Woody of the UNC Flow Cytometry Core for their expert help with FACS sorting. Research reported in this publication and related to FACS sorting was supported in part by the North Carolina Biotech Center Institutional Support Grant 2012-IDG-1006. We thank the UNC High Throughput Sequencing core (HTSF) for sequencing. The above cores affiliated to UNC Lineberger Comprehensive Cancer Center are supported in part by P30 CA016086 Cancer Center Core Support Grant to the UNC Lineberger Comprehensive Cancer Center. The UNC Animal Models Core, and in particular, the expertise of Dale Cowley, for creating the *Tet2^H1795R^* (*Tet2^CD^*) knock-in mice using the CRISPR/Cas9 system. We thank the Duke University School of Medicine for the use of Sequencing and Genomic Technologies Shared Resource, which provided sequencing services. We gratefully acknowledge Professor Anjana Rao (La Jolla Institute for Allergy and Immunology) for the kind gift of *Tet3* ^flx/flx^ CD4Cre mice. We thank the NIH tetramer core for generously providing aGalactosyl-Ceramide loaded mouse CD1d tetramers. 

## Conflict of interest

The authors declare that the research was conducted in the absence of any commercial or financial relationships that could be construed as a potential conflict of interest.

## Publisher’s note

All claims expressed in this article are solely those of the authors and do not necessarily represent those of their affiliated organizations, or those of the publisher, the editors and the reviewers. Any product that may be evaluated in this article, or claim that may be made by its manufacturer, is not guaranteed or endorsed by the publisher.

## Supplementary material

Supplementary Figure 1
*Tet2* and *Tet3* are upregulated during CD4 SP cell maturation and are targets of Th-POK. **(A)** Gene expression [from (Chopp et al., 2020)] of TET proteins in wild type (WT, *in pink*) mature CD4 SP cells and WT immature CD4 SP cells (*in yellow*). Genome browser snapshots of Th-POK binding [by Th-POK ChIP-seq, (Chopp et al., 2020)] across the loci of *Tet* genes: **(B)** *Tet1*, **(C)** *Tet2* and **(D)** *Tet3*. The arrows indicate the direction of transcription. **(E)** Schematic representation of CD4 SP cell maturation. The lineage specifying transcription factor Th-POK drives the maturation of CD4 SP immature to mature CD4 SP cells. During the process *Tet1*, *Tet2* and mainly *Tet3* are upregulated.

Supplementary Figure 2 Sorting strategy and purity assessment of control (wild type) and *Tet2/3* DKO CD4 SP cells. CD24^hi^ cells were depleted by magnetic bead isolation and untouched CD24^low^ cells were stained for TCRb^PERCPCy5.5^, aGalCerCd1d^PE^, CD4^AF488^ and CD8^APC^. Dead cells were excluded using a fixable viability dye. Live cells aGalCerCd1d-TCRb+ CD8-CD4+ were sorted. Cells were evaluated for purity post sorting. The same strategy was applied for both control and *Tet2/3* DKO CD4 SP cells.

Supplementary Figure 3 RNA-seq data comparison of thymic CD4 SP cells. Dissimilarity matrix of RNA-seq datasets of thymic CD4 SP cells. 3 biological replicates (BR) of control (*wild type*, WT) CD4 SP and 2 BR of *Tet2/3* DKO RNA-seq samples.

Supplementary Figure 4 Reduced Gata3 expression in CD4 SP cells. **(A)** Graph depicting *Gata3* gene expression in WT (*gray*) and *Tet2/3* DKO (*purple*) CD4 SP cells (RNA-seq data, generated in this study). While no statistically significant difference was detected, there was a trend for reduced gene expression. **(B)** Representative histogram comparing the protein expression levels of GATA3 in wild type (*gray*) and *Tet2/3* DKO (*purple*) CD4 SP cells evaluated by flow cytometry. **(C)** Median Fluorescence Intensity (MFI) of GATA3 expression in CD4 SP cells as evaluated in **(B)**. Each dot represents a mouse. WT n=7 (in black), *Tet2/3* DKO n=6 (in purple). For each genotype male and female mice were analyzed with comparable findings. 3 independent experiments were performed. ** (p=0.0076), unpaired t test. Horizontal lines indicate the mean (s.e.m.).

Supplementary Figure 5
**(A)** Unprocessed western blots related to **Figures 4D, 4E**. **(B)** Unprocessed western blots related to Supplementary Data **Figure 7A**. The markers are indicated.

Supplementary Figure 6 Phenotypic characterization of thymic development in *Tet2^CD^* mice. **(A)** Plot summarizing the total number of thymocytes in control wild type (n=5) and *Tet2* ^CD^ mice (n=5). **(B)** Representative flow cytometry plots assessing aGalactosylseramide (aGalCer) loaded CD1d tetramer binding and TCRb expression in thymocytes isolated from control and *Tet2* ^CD^ mice. The aGalCer-CD1d tetramer+ TCRb+ cells are iNKT cells. **(C)** Frequency of thymic iNKT, identified as described in **(B)**, in control (shown in black) and *Tet2* ^CD^ mice (depicted in light blue). **(D)** Absolute numbers of thymic iNKT cells in control (in black) and *Tet2* ^CD^ mice. **(E)** Representative flow cytometry plots evaluating CD4 and CD8 expression in the surface of thymocytes (excluding iNKT cells) isolated from control (wild type), and *Tet2* ^CD^ mice. **(F)** Frequency of thymic subsets identified based on CD4 and CD8 expression, as described in **(E)** in control (in black) and *Tet2* ^CD^ mice. Note that in the plot depicting frequency of DP cells the y axis shows values ranging from 80-86. 3 independent experiments were performed. Each dot represents a mouse.WT n=5 (in black), *Tet2* ^CD^ n=5 (in light blue). Horizontal lines indicate the mean (s.e.m.). No statistically significant difference was identified by unpaired t test.

Supplementary Figure 7 Assessing TET2 expression in *Tet2* ^CD^ *Tet3* KO CD4 SP cells. **(A)** Western blot depicting TET2 expression in extracts from sorted CD4 SP cells isolated from control (WT), and *Tet2* ^CD^ *Tet3 *KO mice. GAPDH was used as loading control. Data from one representative experiment out of 4 are shown. **(B)** Quantification of western blots showing expression of TET2. The number of mice evaluated for each genotype is shown in parenthesis. Control (WT) n=4 (2 male and 2 female), *Tet2* ^CD^ *Tet3 *KO n=4 (2 male and 2 female).
