# Supplementary figures and images for: TET proteins regulate T cell and iNKT cell lineage specification in a TET2 catalytic dependent manner
Source: Front Immunol. 2022 Aug 5;13:940995. doi: 10.3389/fimmu.2022.940995 (PMC9389146; doi:10.3389/fimmu.2022.940995)

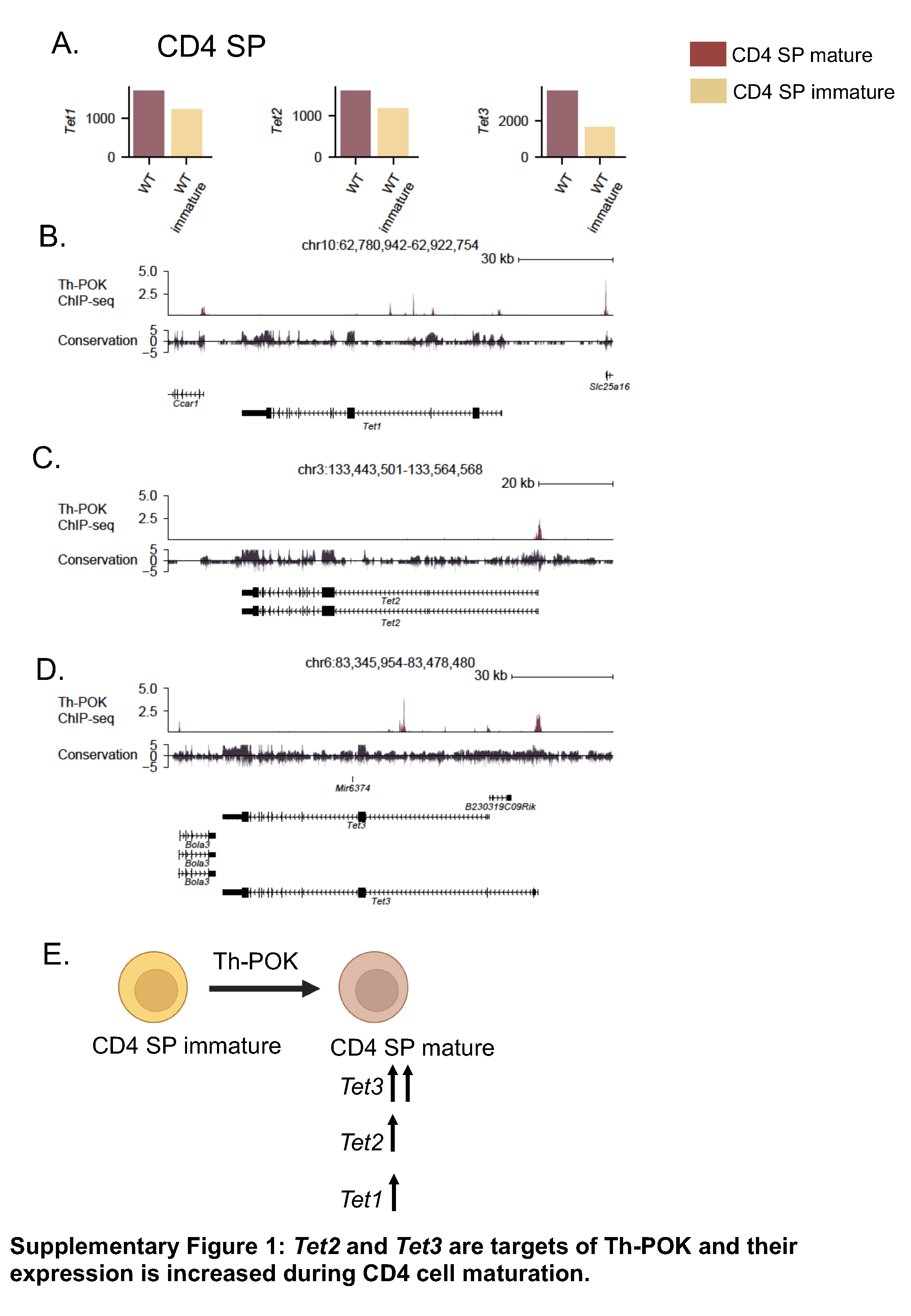

Supplement: Supplementary Figure 1 — Tet2 and Tet3 are upregulated during CD4 SP cell maturation and are targets of Th-POK. (A) Gene expression [from (Chopp et al., 2020)] of TET proteins in wild type (WT, in pink) mature CD4 SP cells and WT immature CD4 SP cells (in yellow). Genome browser snapshots of Th-POK binding [by Th-POK ChIP-seq, (Chopp et al., 2020)] across the loci of Tet genes: (B) Tet1, (C) Tet2 and (D) Tet3. The arrows indicate the direction of transcription. (E) Schematic representation of CD4 SP cell maturation. The lineage specifying transcription factor Th-POK drives the maturation of CD4 SP immature to mature CD4 SP cells. During the process Tet1, Tet2 and mainly Tet3 are upregulated. [file Image_1.tiff]

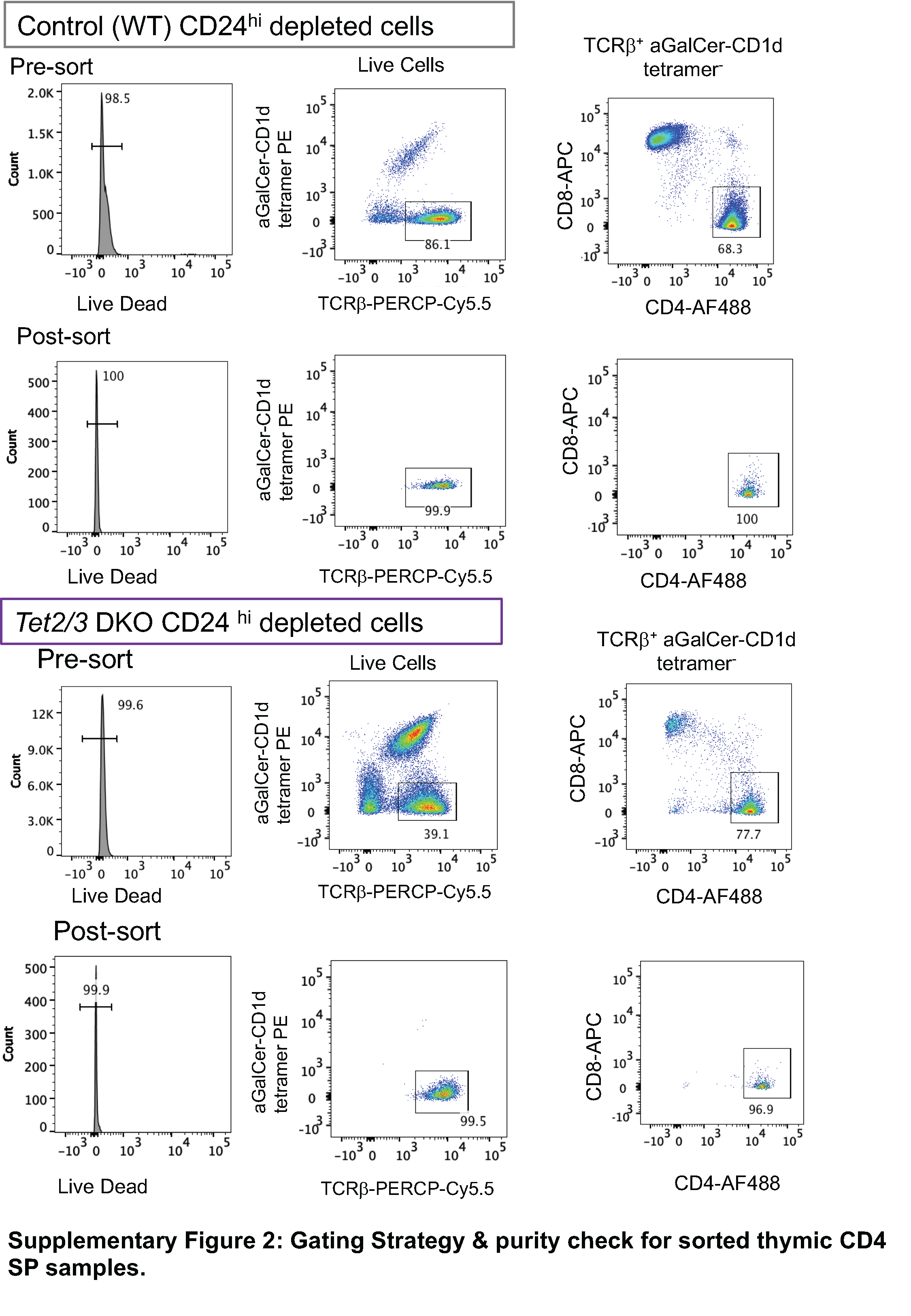

Supplement: Supplementary Figure 2 — Sorting strategy and purity assessment of control (wild type) and Tet2/3 DKO CD4 SP cells. CD24hi cells were depleted by magnetic bead isolation and untouched CD24low cells were stained for TCRbPERCPCy5.5, aGalCerCd1dPE, CD4AF488 and CD8APC. Dead cells were excluded using a fixable viability dye. Live cells aGalCerCd1d-TCRb+ CD8-CD4+ were sorted. Cells were evaluated for purity post sorting. The same strategy was applied for both control and Tet2/3 DKO CD4 SP cells. [file Image_2.tiff]

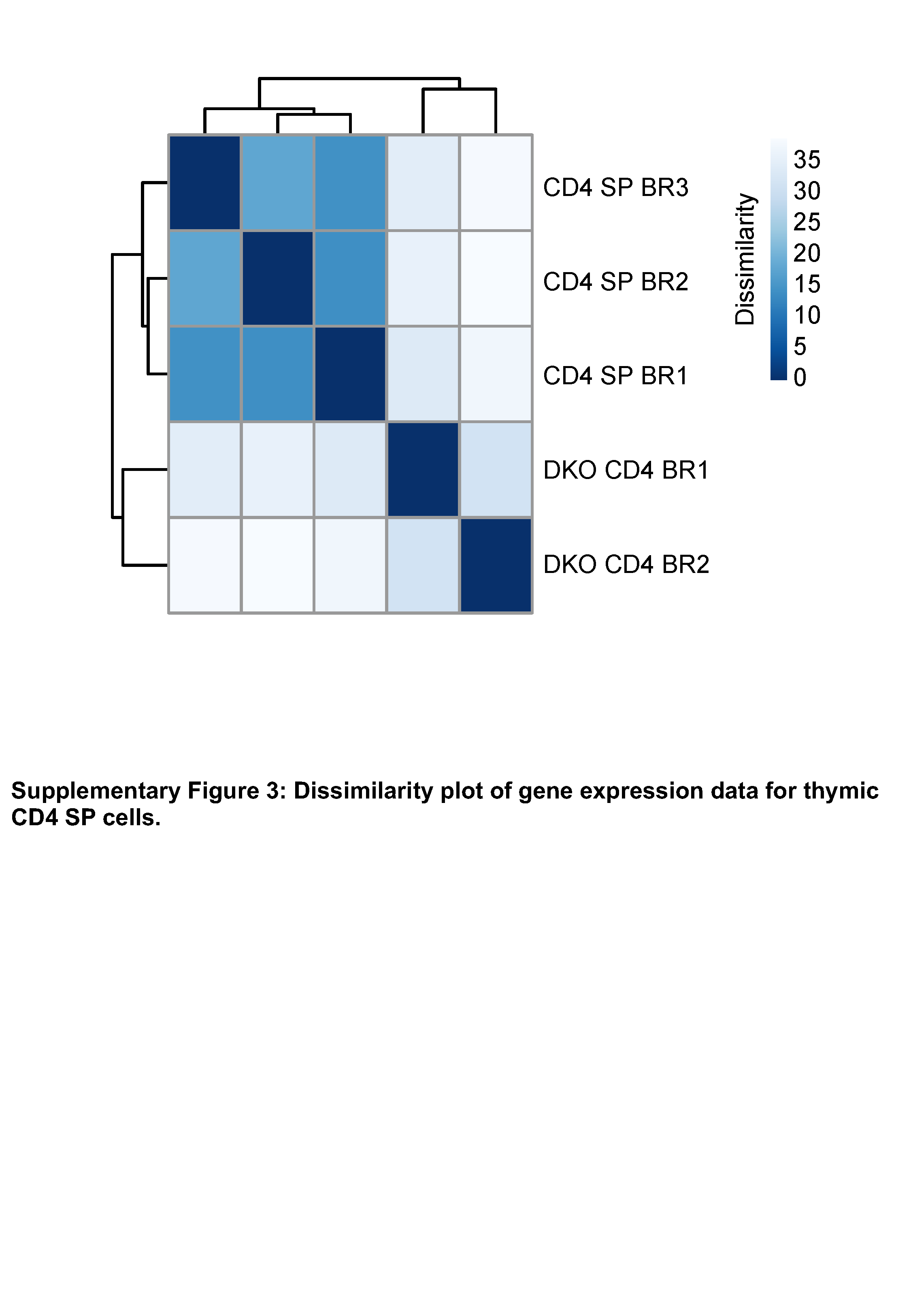

Supplement: Supplementary Figure 3 — RNA-seq data comparison of thymic CD4 SP cells. Dissimilarity matrix of RNA-seq datasets of thymic CD4 SP cells. 3 biological replicates (BR) of control (wild type, WT) CD4 SP and 2 BR of Tet2/3 DKO RNA-seq samples. [file Image_3.tiff]

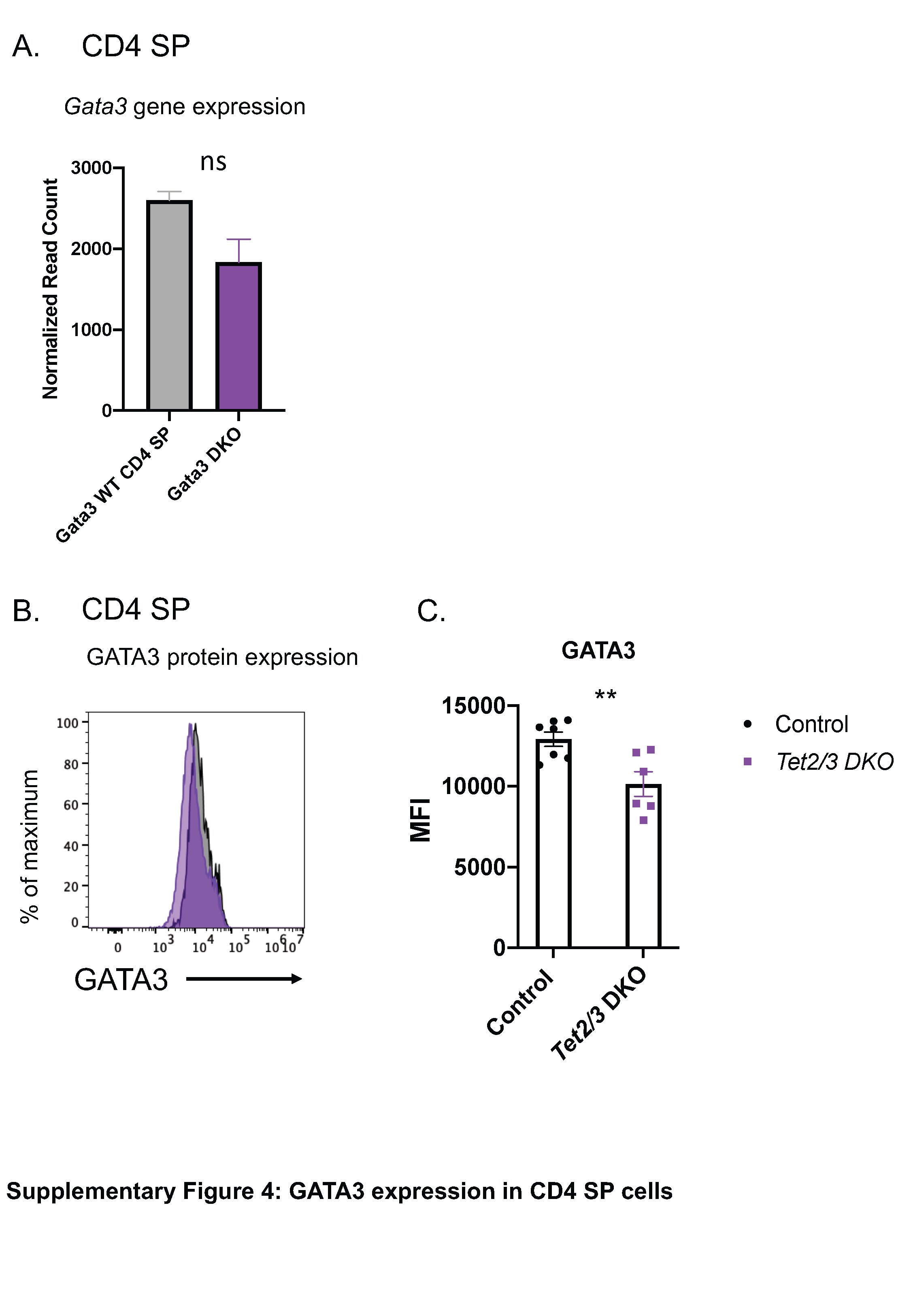

Supplement: Supplementary Figure 4 — Reduced Gata3 expression in CD4 SP cells. (A) Graph depicting Gata3 gene expression in WT (gray) and Tet2/3 DKO (purple) CD4 SP cells (RNA-seq data, generated in this study). While no statistically significant difference was detected, there was a trend for reduced gene expression. (B) Representative histogram comparing the protein expression levels of GATA3 in wild type (gray) and Tet2/3 DKO (purple) CD4 SP cells evaluated by flow cytometry. (C) Median Fluorescence Intensity (MFI) of GATA3 expression in CD4 SP cells as evaluated in (B). Each dot represents a mouse. WT n=7 (in black), Tet2/3 DKO n=6 (in purple). For each genotype male and female mice were analyzed with comparable findings. 3 independent experiments were performed. ** (p=0.0076), unpaired t test. Horizontal lines indicate the mean (s.e.m.). [file Image_4.tiff]

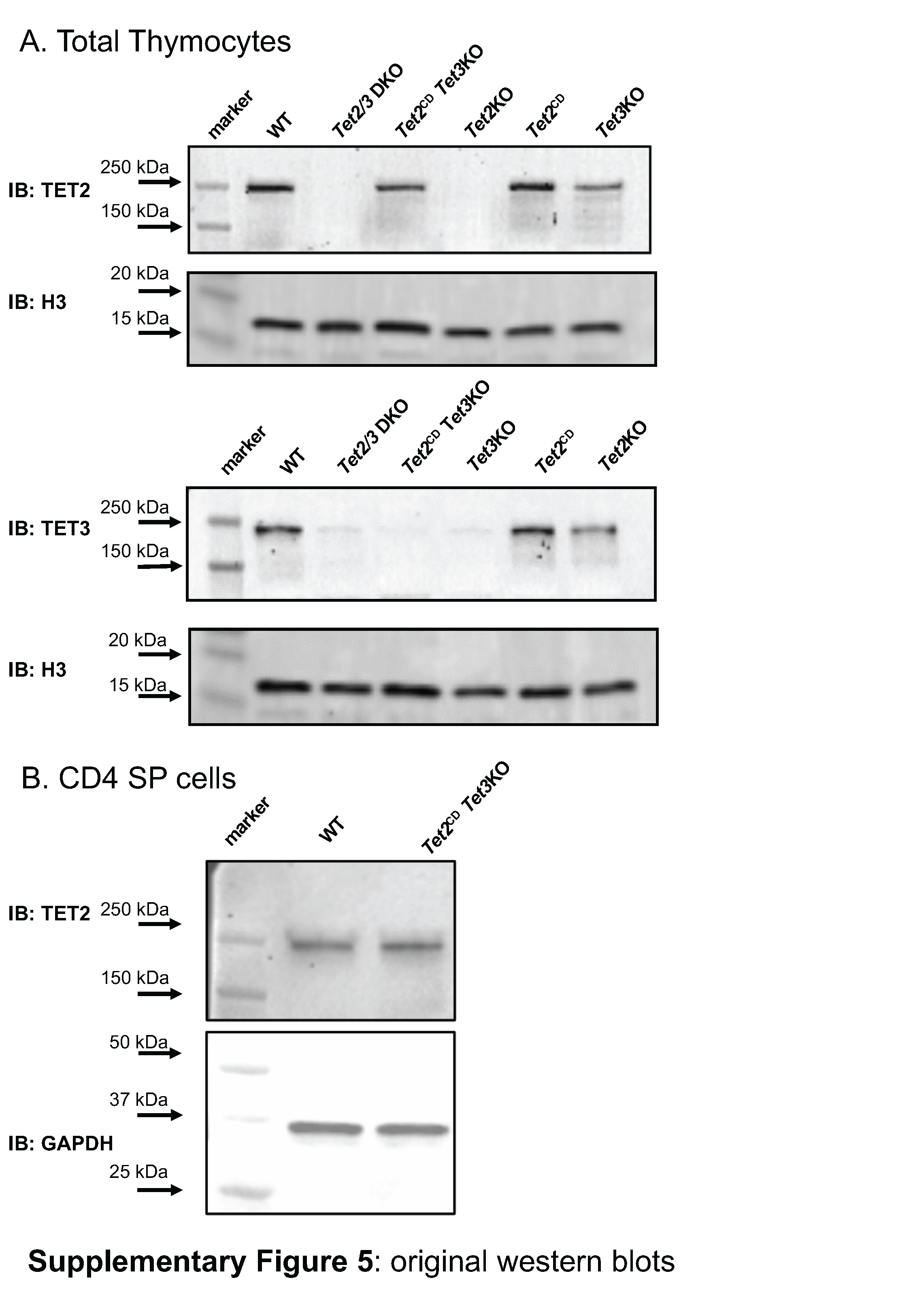

Supplement: Supplementary Figure 5 — (A) Unprocessed western blots related to Figures 4D, 4E . (B) Unprocessed western blots related to Supplementary Data Figure 7A . The markers are indicated. [file Image_5.tiff]

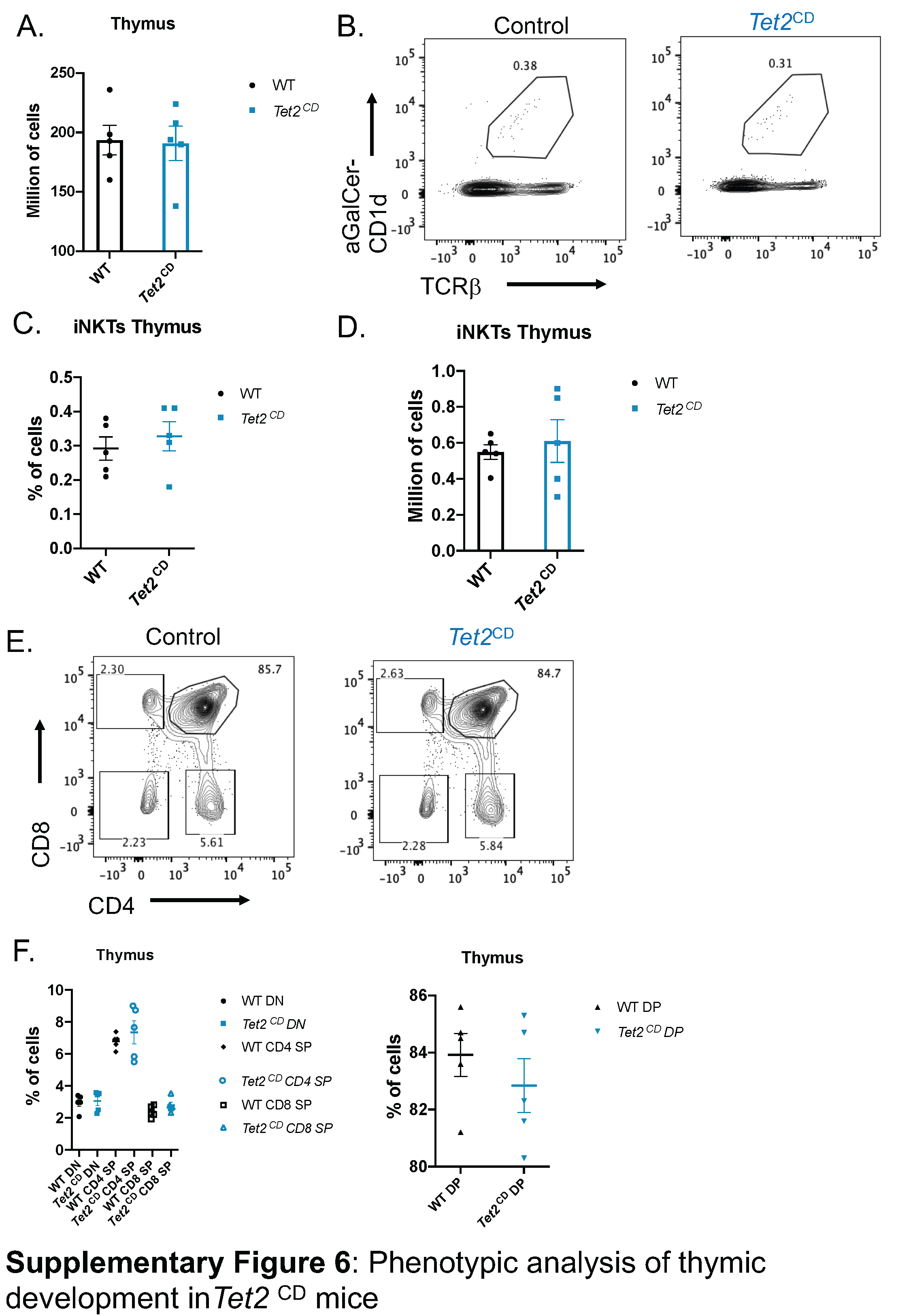

Supplement: Supplementary Figure 6 — Phenotypic characterization of thymic development in Tet2CD mice. (A) Plot summarizing the total number of thymocytes in control wild type (n=5) and Tet2 CD mice (n=5). (B) Representative flow cytometry plots assessing aGalactosylseramide (aGalCer) loaded CD1d tetramer binding and TCRb expression in thymocytes isolated from control and Tet2 CD mice. The aGalCer-CD1d tetramer+ TCRb+ cells are iNKT cells. (C) Frequency of thymic iNKT, identified as described in (B), in control (shown in black) and Tet2 CD mice (depicted in light blue). (D) Absolute numbers of thymic iNKT cells in control (in black) and Tet2 CD mice. (E) Representative flow cytometry plots evaluating CD4 and CD8 expression in the surface of thymocytes (excluding iNKT cells) isolated from control (wild type), and Tet2 CD mice. (F) Frequency of thymic subsets identified based on CD4 and CD8 expression, as described in (E) in control (in black) and Tet2 CD mice. Note that in the plot depicting frequency of DP cells the y axis shows values ranging from 80-86. 3 independent experiments were performed. Each dot represents a mouse.WT n=5 (in black), Tet2 CD n=5 (in light blue). Horizontal lines indicate the mean (s.e.m.). No statistically significant difference was identified by unpaired t test. [file Image_6.tiff]

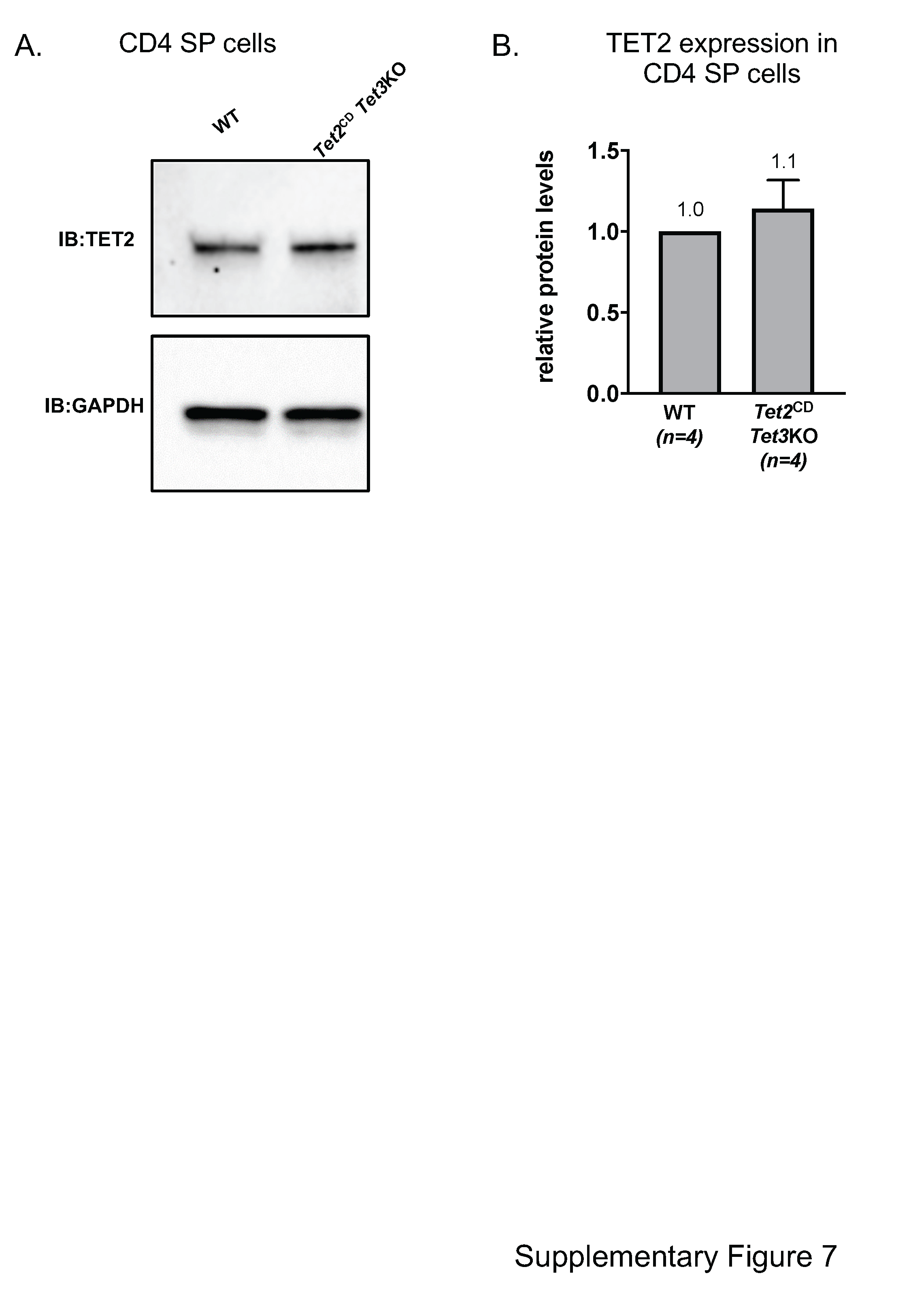

Supplement: Supplementary Figure 7 — Assessing TET2 expression in Tet2 CD Tet3 KO CD4 SP cells. (A) Western blot depicting TET2 expression in extracts from sorted CD4 SP cells isolated from control (WT), and Tet2 CD Tet3 KO mice. GAPDH was used as loading control. Data from one representative experiment out of 4 are shown. (B) Quantification of western blots showing expression of TET2. The number of mice evaluated for each genotype is shown in parenthesis. Control (WT) n=4 (2 male and 2 female), Tet2 CD Tet3 KO n=4 (2 male and 2 female). [file Image_7.tiff]
